# Effects of Mind–Body Exercise on Bone Health in Perimenopausal Women: A Systematic Review and Three-Level Meta-Analysis

**DOI:** 10.3390/life16091389

**Published:** 2026-08-23

**Authors:** Zhuo Zeng, Chengyu Zhou, Lin Luo, Shuaihao Zhao, Xusong Dong, Wenhui Yin, Wenyan Yin, Dongxu Huang, Haoqiang Shi, Haoran Li, Yongmin Xie, Aiguo Zhou, Chengyi Zhang

**Affiliations:** 1 Strength and Conditioning Training College, Beijing Sports University, Beijing 100084, China; 2024210137@bsu.edu.cn (Z.Z.); chengyuzhou0102@gmail.com (C.Z.); 2023012202@bsu.edu.cn (X.D.); dongxuhuang1027@163.com (D.H.); m15966507473@163.com (H.S.); marslee_24@163.com (H.L.); xieyongminbsu@163.com (Y.X.); aiguozhou@126.com (A.Z.); 2 Department of Physical Education, North China Electric Power University, Beijing 102206, China; luolin516@126.com; 3 Sports Medicine and Rehabilitation College, Beijing Sport University, Beijing 100084, China; 2024210369@bsu.edu.cn; 4 Division of sports Science and Physical Education, Tsinghua University, Beijing 100084, China; yinwh24@mails.tsinghua.edu.cn; 5 School of Physical Education and Health, East China Normal University, Shanghai 200241, China; yinwenyan921@163.com; 6 China Basketball College, Beijing Sport University, Beijing 100084, China

**Keywords:** mind–body exercise, perimenopause, bone mineral density, female

## Abstract

The perimenopausal phase represents a critical window for early intervention against accelerated bone loss, highlighting an urgent need for safe and accessible non-pharmacological strategies. Although mind–body exercises are widely recommended for healthy aging, their specific structural and metabolic impacts on the perimenopausal skeleton yield conflicting results and remain poorly understood. To address this gap, this systematic review and three-level meta-analysis evaluated the effects of mind–body modalities on bone health in perimenopausal women, utilizing this model to account for statistical dependencies among multiple effect sizes within individual studies. Quantitative synthesis suggested that mind–body exercise may offer modest structural benefits, indicated by positive effects on bone mineral density (SMD = 0.55, *p* < 0.01) and bone mineral content (SMD = 1.63, *p* < 0.01); however, these findings must be interpreted with caution as the accompanying certainty of evidence is low to very low. Conversely, these structural adaptations were not accompanied by stable alterations in bone turnover markers, which remained consistently unchanged (SMD = −0.10, *p* > 0.05). Furthermore, bone mineral metabolism was not statistically significant in the primary analysis, with a confidence interval that included the null (SMD = 0.90, *p* = 0.079) and demonstrated limited stability across robustness checks. Assessed via the GRADE framework, this overall very low certainty was primarily due to risk-of-bias concerns, inconsistency, imprecision, suspected publication bias, and clinical heterogeneity. Consequently, given the current evidence limitations, mind–body exercise is best conceptualized as a supportive lifestyle component of a healthy aging trajectory rather than a potent osteogenic therapy. It offers a holistic preventative approach that might support bone health while concurrently supporting overall musculoskeletal resilience, though firm clinical recommendations cannot yet be made. Future well-powered trials must adopt rigorous designs with precise endocrine staging to better clarify the underlying mechanisms of skeletal adaptation.

## 1. Introduction

Perimenopause is increasingly recognized as a critical window for preserving skeletal health rather than merely a transitional stage before menopause [1]. During this period, menstrual irregularity, declining ovarian function, and pronounced estrogen fluctuations may disturb the balance between bone formation and bone resorption, making women more vulnerable to accelerated bone loss. Longitudinal studies have shown that bone loss tends to increase around the menopausal transition, particularly at clinically important skeletal sites such as the lumbar spine and femoral neck [2]. Because lower bone mineral density (BMD) is closely associated with later skeletal fragility and fracture risk [3,4], these findings highlight the importance of shifting bone health management from postmenopausal treatment toward earlier prevention during the perimenopausal period, when bone loss is already emerging but may still be modifiable.

Given this early vulnerability, exercise-based interventions have attracted increasing attention as feasible non-pharmacological approaches for skeletal health management in perimenopausal women [5,6]. However, because perimenopausal women differ in physical function, joint stability, and adherence, high-impact or high-intensity exercise may not be universally suitable. In this context, mind–body exercises (e.g., Tai Chi, Baduanjin, Qigong, yoga, Pilates, Wuqinxi, and Yijinjing) have emerged as highly accessible and sustainable alternatives [7,8]. By integrating low-impact movement with breathing regulation and mental focus, these inexpensive modalities not only provide gentle mechanical loading but also concurrently enhance neuromuscular control, balance, and postural stability [9]. Therefore, they represent a practical adjunctive option for bone health management that warrants further evaluation during the perimenopausal period.

Consistent with this rationale, clinical studies have increasingly examined whether mind–body exercise promotes favorable skeletal changes in women [10]. While some trials report potential benefits for bone mineral density (BMD) at weight-bearing sites, others yield limited or non-significant effects [11,12]. Furthermore, previous evidence syntheses provide only partial answers because they predominantly target postmenopausal women, osteoporosis populations, or single exercise modalities (e.g., focusing solely on Tai Chi), leaving the specific effects on perimenopausal women unclear [13,14,15,16,17]. Available findings also exhibit substantial heterogeneity across session duration, training frequencies, skeletal measurement sites, and outcome types [14]. Consequently, current evidence remains suggestive rather than definitive.

To address these limitations, a focused and methodologically robust synthesis is needed. Studies in this field frequently report multiple skeletal sites, multiple bone health outcomes, or multiple time points, resulting in several correlated effect sizes within the same study. Treating these effect sizes as independent in a conventional two-level meta-analysis may underestimate standard errors, whereas selecting only one effect size from each study leads to information loss [18]. Although multivariate meta-analysis is a traditional solution for correlated outcomes, its application is severely hindered because primary trials rarely report the required within-study correlation matrices. A three-level meta-analytic model provides a more robust and practical alternative; it accounts for the hierarchical data structure (effect sizes nested within studies) to manage complex dependencies without demanding unavailable covariance data. Therefore, the present study aims to systematically evaluate the effects of mind–body exercise on bone health in perimenopausal women using a three-level meta-analysis. BMD will be considered the primary outcome, while bone mineral content (BMC), bone mineral metabolism (BMM), and bone turnover markers (BTMs) will be analyzed as secondary outcomes. This review is intended to provide a methodologically appropriate synthesis of current evidence to inform future high-quality interventions.

## 2. Materials and Methods

This systematic review and three-level meta-analysis were conducted and reported in accordance with the Preferred Reporting Items for Systematic Reviews and Meta-Analyses 2020 statement (PRISMA 2020) [19]. The completed PRISMA 2020 checklist is provided in Supplementary Materials Table S1. PROSPERO registration was completed on 29 June 2026 (CRD420261436898), at which point the review had progressed to study screening. No changes were made to the registered protocol during the subsequent conduct of the review.

### 2.1. Information Sources and Search Strategy

A systematic literature search was conducted in PubMed, Web of Science, Cochrane Library, MEDLINE, SPORTDiscus, Scopus, WanFang, VIP, and Chinese Medical Journals Full-text from database inception to 23 May 2026. No language restrictions were applied. An updated search was conducted on 15 August 2026 and no additional eligible studies were identified.

The search strategy combined terms related to the target population and intervention. Population-related terms included perimenopause, perimenopausal women, menopausal transition, and related synonyms. Intervention-related terms included mind–body exercise and specific exercise modalities, such as Tai Chi, Taijiquan, Baduanjin, Qigong, yoga, Pilates, Wuqinxi, and Yijinjing. Corresponding Chinese-language terms were used in the Chinese databases. In addition, the reference lists of relevant studies and reviews were screened to identify additional eligible records, and forward citation searching of the included studies was conducted as a supplementary search method. No separate systematic search of clinical trial registries was performed. Full-text theses and dissertations identified through this process were assessed using the same predefined eligibility criteria as journal articles. The complete, detailed search strategy is available in Appendix A.

### 2.2. Selection Process

Following the removal of duplicates using EndNote X9 (Clarivate, Philadelphia, PA, USA), the titles and abstracts of all unique records were screened independently by two reviewers (Z.Z. and X.S.D). This screening was based on the predefined eligibility criteria. The same two reviewers then independently assessed the full texts of potentially eligible studies for final inclusion. At both screening stages, any disagreements were resolved through discussion or, if necessary, adjudicated by a third reviewer (W.H.Y).

### 2.3. Eligibility Criteria

Eligibility requirements were structured around the five PICOS domains: population, intervention, comparator, outcome, and study design. Eligible studies included perimenopausal women, used a single mind–body exercise intervention, and reported at least one bone health-related outcome with sufficient data for effect size calculation. Studies involving combined interventions, inappropriate populations, unavailable outcome data, or unsuitable study designs were excluded. This strict exclusion of combined treatments was necessary to eliminate the confounding effects of potent bone-modulating agents (such as calcium or vitamin D) and strictly isolate the independent impacts of mind–body exercise on the skeleton. For multi-arm trials, only intervention arms that met the predefined criteria for mind–body exercise, together with the corresponding eligible control group, were included in the quantitative synthesis. Study eligibility criteria are summarized in Table 1.

### 2.4. Data Extraction

Study information was entered into a Microsoft Excel extraction form that had been prepared in advance of the full-text assessment. Y.W.Y. and W.H.Y. independently recorded bibliographic information, methodological features, participant profiles, intervention characteristics, and outcome data for each eligible study. D.X.H. then cross-checked the completed dataset against the original reports. Differences identified during verification were settled by discussion, and unresolved cases were referred to Y.M.X. for further review.

For studies with missing numerical data or outcomes reported only in graphical form, the corresponding authors were contacted to request additional information. When no response was obtained, data were extracted from figures using WebPlotDigitizer (version 4.1, Ankit Rohatgi, Belmont, CA, USA). Data were extracted according to the analysis population reported in the original studies, with intention-to-treat data used when available and completer or available case data used otherwise. Studies were excluded from the corresponding meta-analysis when the required data remained unavailable.

### 2.5. Data Processing

For the quantitative analysis, the mean, standard deviation (SD), and sample size for each group were extracted at both pre- and post-intervention. These values were used to calculate the mean change from pre-test and the corresponding SD for effect size pooling.

The mean change, denoted as MD_diff_, was calculated as the post-intervention mean minus the baseline mean, as shown in Equation (1):

(1) $${MD}_{diff\;}=\;M_{post}\;-\;M_{pre}$$

where MD_diff_ represents the raw mean difference, M_post_ denotes the reported post-intervention mean, and M_pre_ denotes the reported pre-intervention mean.

When standard deviations were not directly reported but confidence intervals were available, SDs were derived using Equation (2):

(2) $$SD=\sqrt{N}\;\frac{{CI}_{high}-\;{CI}_{low}}{2t}$$

where SD denotes the standard deviation; N denotes the group sample size; CI_high_ and CI_low_ represent the upper and lower limits of the confidence interval, respectively; and t refers to the t-distribution value, with N − 1 degrees of freedom at the corresponding confidence level.

The SD_diff_ was calculated using Equation (3) provided in the Cochrane Handbook [20]:

(3) $${SD}_{diff}=\sqrt{{{SD}_{pre}}^{2}+{{SD}_{post}}^{2}-2r\times {SD}_{pre}\times {SD}_{post}}$$

where SD_diff_ denotes the standard deviation of the change score, SD_pre_ denotes the pre-intervention standard deviation, and SD_post_ denotes the post-intervention standard deviation.

Because the included studies did not report Pearson’s correlation coefficients (r) between pre- and post-intervention values, a correlation coefficient of 0.5 was assumed according to the Cochrane Handbook [21]. Sensitivity analyses using alternative r values ranging from 0.3 to 0.7 showed that the main pooled estimates remained stable. Equation (4) was provided as follows:

(4) $$r=\frac{{{SD}_{pre}}^{2}+{{SD}_{post}}^{2}-{{SD}_{change}}^{2}}{2\times {SD}_{pre}\times {SD}_{post}}$$

### 2.6. Risk of Bias Assessment

Risk of bias was evaluated independently by two reviewers [X.S.D and H.Q.S] using the Cochrane Risk of Bias 2 (RoB 2) tool. The assessment considered potential bias arising from the randomization process, deviations from intended interventions, missing outcome data, outcome measurement, selection of reported results, and other relevant sources [22]. Any disagreements were resolved through discussion, and unresolved discrepancies were adjudicated by a third reviewer [C.Y.Z].

### 2.7. Statistical Analysis

All statistical analyses were conducted in R (version 4.5.0, R Foundation for Statistical Computing, Vienna, Austria) [23].

#### 2.7.1. Overall Analytical Model

Several included studies contributed multiple intervention arms or outcome measures, which may introduce statistical dependence among effect sizes. Under such conditions, a conventional two-level meta-analytic model may underestimate uncertainty and produce overly precise pooled estimates [24]. To address this issue, a three-level random-effects model was applied. This model decomposes total variance into sampling variance at Level 1, within-study variance at Level 2, and between-study variance at Level 3, thereby accounting for both the hierarchical data structure and dependencies among effect sizes [25]. Specifically, Level 2 variance reflects heterogeneity among multiple effect sizes derived from the same study, for example, effects estimated for different skeletal sites or related bone-health outcomes within the same participant sample. In contrast, Level 3 variance represents heterogeneity in the underlying effects across independent studies. Thus, a larger Level 2 variance component indicates that variability is primarily attributable to differences among effect sizes within studies, whereas a larger Level 3 component indicates greater variability in effects between studies.

Cluster-robust variance estimation (CRVE) with small-sample correction was additionally used to account for dependence among effect sizes originating from the same study. Specifically, the CR2 adjustment proposed by Bell and McCaffrey [26] was applied to accommodate correlated outcomes and obtain small-sample-adjusted standard errors and confidence intervals [27,28]. Rather than selecting, averaging, or excluding dependent effect sizes, all eligible effect sizes were retained to preserve available information and statistical power [24]. Model parameters were primarily estimated using restricted maximum likelihood (REML), with maximum likelihood (ML) estimation conducted as a robustness check [29].

#### 2.7.2. Effect Size and Heterogeneity Assessment

The standardized mean difference (SMD) was used as the summary effect measure and expressed as Hedges’ g to adjust for small-sample bias. The magnitude of effect sizes was categorized as trivial (<0.2), small (0.2–0.5), moderate (0.5–0.8), or large (>0.8) [30]. Statistical heterogeneity across studies was examined using Cochrane’s Q, I^2^, τ^2^, and τ. In addition, 95% confidence intervals (CIs) and prediction intervals (PIs) were calculated to describe the uncertainty and expected dispersion of effects [31]. I^2^ was used as the main indicator of heterogeneity and interpreted as low (0–25%), moderate (25–50%), substantial (50–75%), or considerable (75–100%) [21].

#### 2.7.3. Moderator and Sensitivity Analyses

Potential sources of heterogeneity were explored using subgroup analyses and meta-regression when overall heterogeneity exceeded 25% based on I^2^ [32]. Given the limited number of independent studies, these subgroup and meta-regression analyses were considered exploratory and hypothesis-generating rather than confirmatory, and their findings were therefore interpreted with caution. Potential moderators were selected separately for each outcome according to the pre-specified study protocol, theoretical relevance, and data availability. For BMD, subgroup analyses were conducted according to body part, session duration, training frequency, and mind–body exercise type, while intervention period was additionally examined using meta-regression. For BMC, subgroup analyses were conducted according to body part, session duration, and training frequency. For BMM, serum calcium, magnesium, and phosphorus were operationally grouped to enable quantitative synthesis of related mineral-metabolism outcomes reported across studies. Because these analytes are physiologically distinct and do not directly represent skeletal remodeling, the pooled BMM estimate was treated as an exploratory summary, and subgroup analyses were conducted according to mineral type, training frequency, mind–body exercise type, and session duration. For BTMs, subgroup analyses considered marker type, training frequency, mind–body exercise type, and session duration. For clarity, session duration refers to the duration of each individual exercise session, training frequency refers to the number of exercise sessions per week, and intervention period refers to the total length of the intervention.

Sensitivity analyses were performed using a leave-one-out approach to identify potentially influential studies. Given the small number of independent studies contributing to each outcome, contour-enhanced funnel plots and Egger’s regression test were used only as exploratory assessments of potential small-study effects. Because these methods have limited reliability when few independent studies are available, particularly in the presence of dependent effect sizes, the results were not used to make definitive inferences regarding the presence or absence of publication bias [33].

### 2.8. Certainty of the Evidence

The certainty of evidence for each outcome was assessed using the Grading of Recommendations Assessment, Development, and Evaluation (GRADE) approach and classified as high, moderate, low, or very low [34]. Two reviewers independently performed the GRADE assessment, and any disagreements were resolved through discussion until consensus was reached.

The certainty of evidence was downgraded according to the following criteria: risk of bias, with one level downgraded for some concerns and two levels for high risk [35]; inconsistency, with one level downgraded when moderate heterogeneity was present (I^2^ > 25%) and two levels when heterogeneity was high (I^2^ > 75%) [36]; imprecision, with one level downgraded when the direction of effect was uncertain [37]; and publication bias, with one level downgraded when publication bias could not be reliably excluded based on a comprehensive evaluation incorporating the limited number of included studies (e.g., k < 10), suspected missing evidence, visual assessment of funnel-plot asymmetry, and other contextual considerations, rather than relying solely on the statistical significance of Egger’s test [38].

## 3. Results

### 3.1. Study Selection

A literature search was conducted across nine databases: PubMed, Web of Science, the Cochrane Library, EBSCO-MEDLINE, EBSCO-SPORTDiscus, Scopus, and three Chinese databases: WanFang, VIP, and Chinese Medical Journals Full-text. The initial search, performed on 23 May 2026, identified 6139 publications. After removing 1649 duplicates, 4490 records were screened based on titles and abstracts. Additionally, three studies were identified through other sources. A subsequent update of the literature search on 15 August 2026 yielded no additional eligible studies.

**Figure 1:**
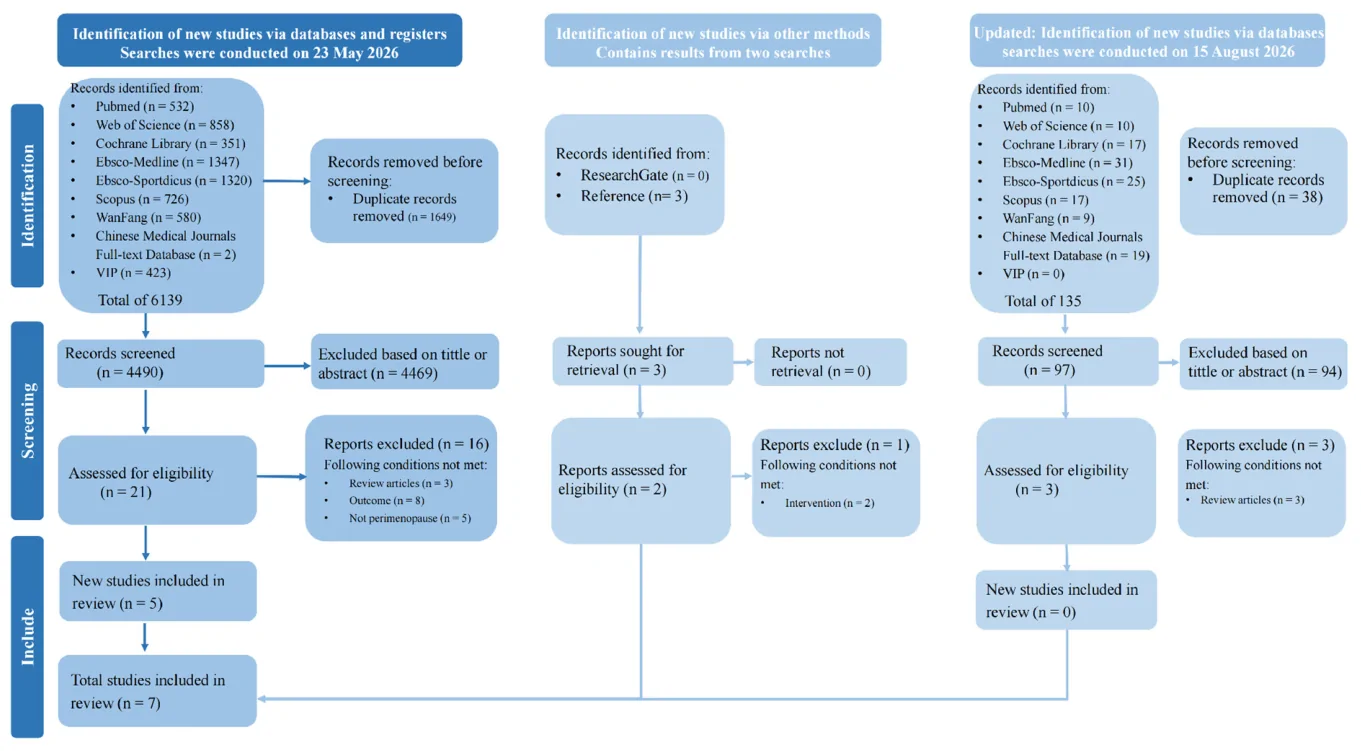
PRISMA flow diagram of study selection process.

After a full-text assessment, a total of seven studies were deemed eligible and included in the final meta-analysis [10,39,40,41,42,43,44]. The detailed study selection process is shown in Figure 1.

### 3.2. Characteristics of Included Studies

All seven included studies were randomized controlled trials (RCTs), comprising a total of 435 participants. Sample sizes within individual study groups ranged from 15 to 40, and participants were between 49.7 and 55.5 years of age. While basic demographics and intervention parameters (e.g., exercise modality, frequency, and duration) were consistently reported across trials, definitions of perimenopause varied considerably across studies. Some studies relied primarily on age, menopausal symptoms, menstrual history, or Kupperman scores, whereas one study incorporated menstrual irregularity together with estradiol and FSH thresholds, and another applied formal STRAW criteria for reproductive staging. Several studies, however, did not provide a standardized operational definition of menopausal stage. This variation in reproductive-stage ascertainment may represent an important source of clinical heterogeneity. In general, intervention characteristics were generally well reported, whereas reproductive-stage ascertainment was less consistent across studies. Baseline characteristics and study-specific definitions of perimenopausal stage are summarized in Appendix B, while funnel plots for exploratory assessment of potential small-study effects for the pooled outcomes are presented in Appendix C.

It is worth noting that alongside well-known modalities like Tai Chi and Hatha yoga, some included studies utilized specific, culturally rooted mind–body interventions. Given the cultural specificity of these interventions, brief descriptions are provided below: Tai Chi Rouli Ball is a modern mind–body exercise that combines traditional Tai Chi principles with racket movements, emphasizing circular, fluid motions, continuous postural shifts, and mind–body coordination to control a sand-filled ball on a racket. Wu Xing Jian Gu Cao (Five-Element Bone-Strengthening Exercise) is a traditional Chinese movement sequence grounded in the Five-Element theory of traditional Chinese medicine, specifically designed to stimulate bone metabolism, improve joint mobility, and enhance postural control through gentle weight-bearing postures.

### 3.3. Results of the Risk of Bias Assessment

**Figure 2:**
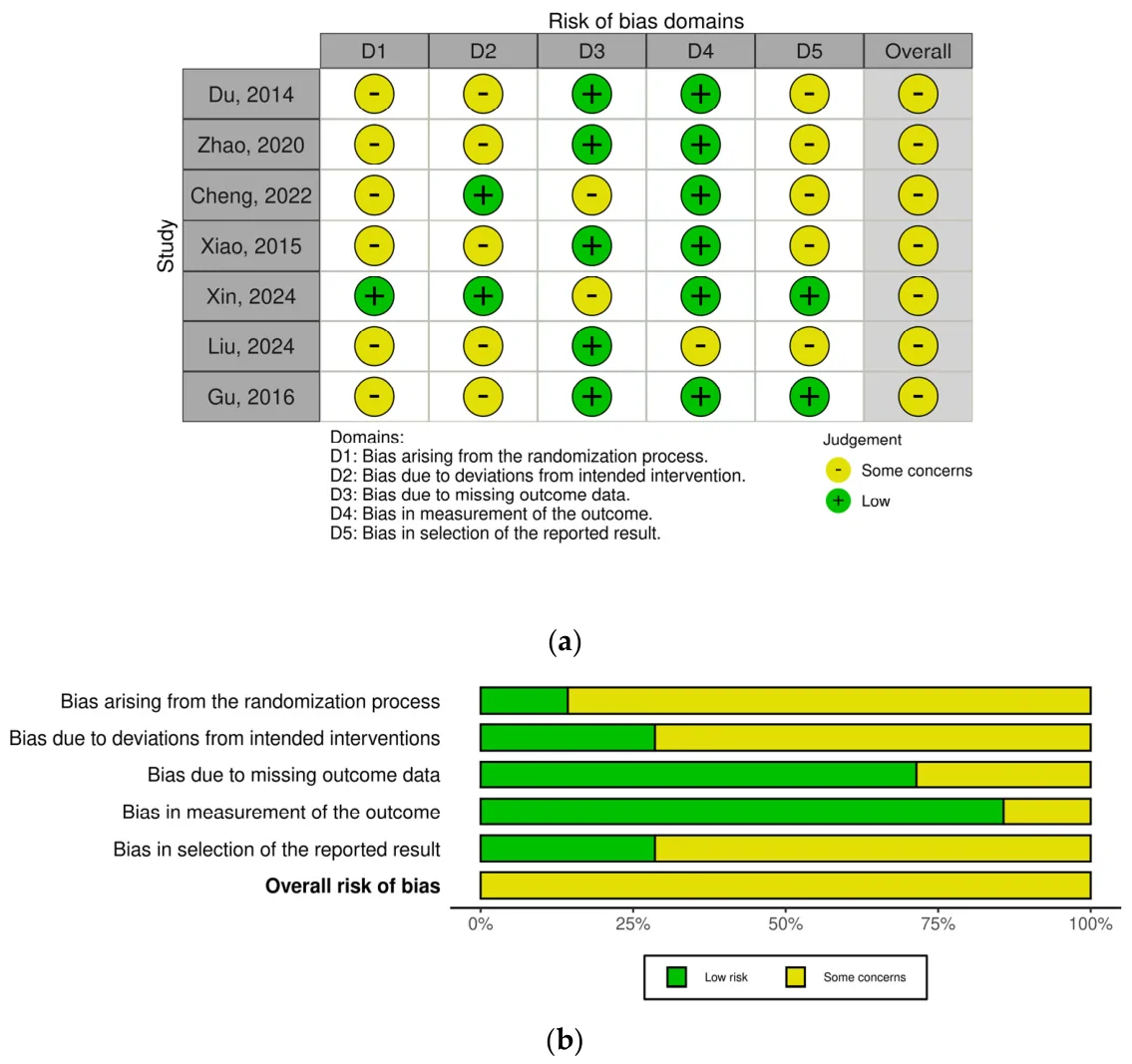
Risk of bias assessment diagram [10,39,40,41,42,43,44]. (**a**) RoB 2 traffic light plot; (**b**) RoB 2 summary plot.

Risk of bias in the included randomized controlled trials was assessed using the RoB 2 tool. Most studies were rated as having “some concerns” overall, with no trials judged at high risk of bias. Domain-level assessment showed that concerns related to the randomization process (Domain 1) were present in approximately 86% of studies. Deviations from intended interventions (Domain 2) raised some concerns in 71% of studies. Missing outcome data (Domain 3) was generally at low risk, with concerns identified in only a small proportion of studies (29%). Outcome measurement (Domain 4) showed some concerns in 14% of studies, while selection of the reported result (Domain 5) was also a frequent source of concern, affecting 71% of studies. These domain-level concerns may reduce confidence in the pooled effect estimates and therefore warrant cautious interpretation. Study-level judgments and their supporting rationales are provided in Supplementary Materials Table S2, with graphical summaries presented in Figure 2a,b.

### 3.4. Meta-Analysis Results

#### 3.4.1. Bone Mineral Density

A three-level meta-analysis was conducted to examine the effect of mind–body exercise on BMD in perimenopausal women. Using restricted maximum likelihood (REML), 37 effect sizes from six studies were included, showing a statistically significant positive effect compared with the control group (k = 37, SMD = 0.55, 95% CI [0.29, 0.80], *p* < 0.01). Heterogeneity was substantial at Level 2 (within-study variance 61.23%) and negligible at Level 3 (between-study variance 0%), with a 95% prediction interval of [−0.30, 1.39]. Because the prediction interval crossed zero, the true effect in a future study conducted under comparable conditions could plausibly be null or negative. Given the limited number of independent studies (*n* = 6), dependence among multiple effect sizes from the same study was further addressed using CR2-adjusted cluster-robust variance estimation. This supplementary analysis yielded a pooled effect of 0.55 (95% CI [0.37, 0.72], Satterthwaite-adjusted df = 4.59, *p* < 0.01), consistent with the primary estimate. However, the small number of independent study clusters limits confidence in the CR2-based inference. Overall, the pooled findings favor mind–body exercise for BMD, but the prediction interval indicates that the effect may not be consistently positive across future comparable studies. The corresponding forest plot is shown in Appendix D.1.

Exploratory subgroup analyses suggested a between-subgroup difference across body regions (*p* = 0.0273), with relatively larger effects observed at the spine and hip compared with other skeletal sites. Given the small number of studies contributing to several subgroup categories, this finding should be regarded as hypothesis-generating. No clear between-group differences were found for intervention session duration, frequency, or exercise type, although all subgroups showed generally positive effects. The detailed results are presented in Table 2.

Meta-regression analyses showed no significant association between intervention period and BMD in either linear (*p* = 0.5314) or non-linear models (quadratic model: *p* = 0.4565; exponential model: *p* = 0.2239), indicating no evidence of a dose–response relationship over time.

#### 3.4.2. Bone Mineral Content

A three-level meta-analysis was performed to evaluate the effect of mind–body exercise on BMC in perimenopausal women. Based on REML, 18 effect sizes from three studies were synthesized, yielding a statistically significant pooled estimate compared with the control group (k = 18, SMD = 1.63, 95% CI [0.86, 2.40], *p* < 0.01). Considerable heterogeneity was observed at Level 2 (within-study heterogeneity, 91.46%), whereas Level 3 heterogeneity was negligible (between-study heterogeneity, 0%), with a wide prediction interval of [−1.28, 4.54]. Because the prediction interval crossed zero, the true effect in a future study conducted under comparable conditions could plausibly be null or negative. Considering the small number of independent studies (*n* = 3) and dependent effect sizes, cluster-robust variance estimation with CR2 correction was additionally used to obtain small-sample-adjusted inferences, yielding the same point estimate but a wider confidence interval (SMD = 1.63, 95% CI [0.64, 2.62], Satterthwaite-adjusted df = 1.99, *p* = 0.02). However, the very small number of study clusters and low effective degrees of freedom limit confidence in this adjusted inference. Taken together, although the pooled estimate favored mind–body exercise, the substantial within-study heterogeneity, wide prediction interval, limited number of independent studies, and very low certainty of evidence according to GRADE preclude concluding that a consistently large benefit would be observed across settings.

Subgroup analyses for BMC were conducted according to body region, intervention session duration, frequency, and exercise type. No significant between-subgroup differences were observed across any subgroup factors (*p* > 0.05). Although effect sizes varied across skeletal sites, no consistent moderator effect was identified. Similar patterns were observed for intervention session duration, frequency, and exercise type, with generally positive but non-differential effects across subgroups. However, several subgroup categories were represented by only one or two independent studies; therefore, these subgroup findings should be regarded as exploratory and hypothesis-generating rather than confirmatory. Collectively, subgroup analyses did not identify significant moderators of the effect of mind–body exercise on BMC. Detailed results are provided in Table 3.

#### 3.4.3. Bone Mineral Metabolism

A multivariate three-level meta-analysis was conducted to examine the effect of mind–body exercise on bone mineral metabolism in perimenopausal women. Based on restricted maximum likelihood (REML), eight effect sizes from three studies were synthesized. The pooled estimate suggested a positive average effect, but this effect was not statistically significant (k = 8, SMD = 0.90, 95% CI [−0.14, 1.92], *p* = 0.079). Heterogeneity was moderate at both Level 2 (I^2^ = 48.69%) and Level 3 (I^2^ = 38.18%). The 95% prediction interval was wide and crossed zero [−1.39, 3.18], indicating that positive, null, or negative effects could plausibly occur in future studies conducted under comparable conditions. Given the limited number of independent studies and the dependence among effect sizes, CR2-adjusted cluster-robust variance estimation was additionally performed as a supplementary analysis. The adjusted analysis yielded the same point estimate (SMD = 0.90), but with a wider 95% CI [−0.99, 2.78] and a non-significant *p*-value (Satterthwaite-adjusted df = 2.00, *p* = 0.177). However, the very small number of independent study clusters and low effective degrees of freedom limit confidence in this adjusted inference. Overall, the evidence for an effect on bone mineral metabolism remains inconclusive.

Subgroup analyses for BMM were conducted according to mineral type, session duration, frequency, and mind–body exercise type. A statistically significant between-subgroup difference was observed for mineral type (*p* = 0.0031), with calcium showing a relatively larger effect compared with magnesium and phosphorus. This finding should be interpreted cautiously as a statistical subgroup signal, given the limited number of studies within each mineral category. No significant subgroup differences were identified for intervention session duration, frequency, or exercise type. Taken together, the findings suggest a non-significant positive trend of mind–body exercise on bone metabolism, accompanied by substantial heterogeneity. See Table 4 for detailed results.

#### 3.4.4. Bone Turnover Markers

A three-level meta-analysis was conducted to examine the effect of mind–body exercise on BTMs in perimenopausal women. Based on REML, seven effect sizes from four studies were synthesized. The pooled effect was not statistically significant (k = 7, SMD = −0.10, 95% CI [−0.53, 0.33], *p* = 0.586). Substantial heterogeneity was observed at Level 2 (I^2^ = 52.57%), whereas Level 3 heterogeneity was negligible (I^2^ = 0%), indicating that heterogeneity was primarily attributable to differences among effect sizes within studies. The 95% prediction interval crossed zero and ranged from −0.95 to 0.75, indicating uncertainty in the distribution of the underlying effects. Given the limited number of independent studies and the dependence among effect sizes, CR2-adjusted cluster-robust variance estimation was additionally performed as a supplementary analysis. The adjusted analysis yielded the same point estimate (SMD = −0.10), with a wider 95% CI [−0.66, 0.46], and remained non-significant (Satterthwaite-adjusted df = 2.42, *p* = 0.57). However, the small number of independent study clusters and low effective degrees of freedom limit confidence in this adjusted inference. Overall, there was no clear evidence of an effect of mind–body exercise on BTMs, with heterogeneity occurring primarily at the within-study level.

Subgroup analyses showed no significant between-subgroup differences across marker type, frequency, exercise type, or session duration (all *p* > 0.05). Effect estimates were generally consistent across subgroups, with no clear evidence of effect modification. However, several subgroup categories were represented by only one or two independent studies; therefore, these subgroup findings should be interpreted cautiously and regarded as exploratory and hypothesis-generating rather than confirmatory. Detailed subgroup results are reported in Table 5.

### 3.5. Sensitivity Analysis

To assess the robustness of the pooled estimates, leave-one-out sensitivity analyses were conducted for all outcomes. For BMD and BMC, the pooled estimates remained relatively stable across iterations. BMD remained statistically significant with minimal variation in effect sizes (SMD: 0.515–0.600), while BMC estimates remained positive (SMD: 1.465–1.859). Overall, the exclusion of any single study did not materially affect these estimates. For BTMs, the pooled estimates remained non-significant across all iterations; however, the direction of the effect fluctuated around zero, with both positive and negative estimates observed (SMD: −0.210 to 0.058). Despite this variation, none of the iterations altered the overall statistical inference, indicating consistent null findings across analyses. The detailed plots are presented in Appendix D.2.

For BMM, the primary analysis was not statistically significant, with the 95% CI including the null (ES = 0.90, 95% CI [−0.14, 1.92], *p* = 0.079). Leave-one-out analyses indicated sensitivity to study composition, with effect estimates varying across iterations. In particular, exclusion of Gu 2016 resulted in a change in statistical significance (ES = 1.321, 95% CI [0.281, 2.362], *p* = 0.01), whereas other leave-one-out scenarios generally yielded non-significant results. These findings suggest limited robustness of the pooled estimate for BMM.

Because the original studies did not report pre-post Pearson’s correlation coefficients, additional sensitivity analyses were conducted by varying the assumed correlation coefficient from r = 0.3 to r = 0.7, with r = 0.5 used in the primary analyses. For BMD and BMC, the pooled effect sizes increased with higher correlation coefficients, but the direction and statistical significance of the effects remained unchanged across all scenarios, indicating stability of the estimates across the assumed correlation values. For BMM, the effect estimates varied slightly with different assumed correlation values; however, the overall conclusion remained unchanged, with results consistently showing a non-significant effect across the examined correlation values. For BTMs, the pooled estimates were stable across all assumed correlation coefficients, with no change in effect direction or statistical significance. Overall, the sensitivity analyses demonstrated that the primary findings were robust to changes in the assumed pre–post correlation coefficient, and the detailed results are summarized in Table 6.

### 3.6. Results of the Certainty of the Evidence

Overall, the certainty of the evidence was very low across all outcomes in Table 7. Downgrading was primarily driven by risk of bias and suspected publication bias (due to the small number of available studies), with inconsistency and imprecision contributing to lower ratings for several outcomes.

## 4. Discussion

This systematic review and three-level meta-analysis found that mind–body exercise was associated with favorable pooled estimates for several bone structural outcomes in perimenopausal women. Statistically significant pooled effects were observed for BMD and BMC; however, the magnitude and consistency of these effects should be interpreted cautiously in light of the substantial heterogeneity, prediction intervals crossing zero, and low to very low certainty of evidence. Findings for BMD and BMC remained broadly similar across leave-one-out sensitivity analyses and variations in the assumed pre–post correlation coefficient. In contrast, BTMs showed no statistically significant effects, although effect estimates fluctuated around zero. BMM was not statistically significant in the primary analysis; however, its results varied across sensitivity analyses, indicating limited stability. Overall, the supplementary and sensitivity analyses produced estimates that were broadly consistent with the primary models, particularly for BMD and BMC, whereas greater uncertainty was observed for BMM. However, the limited number of independent studies constrains the strength of inference from the CR2-adjusted analyses, especially for outcomes informed by only three studies. Accordingly, the CR2 analyses should be regarded as supportive sensitivity checks rather than independent confirmation of robustness, and they do not eliminate the uncertainty arising from heterogeneity, limited study numbers, and the low overall certainty of evidence. Taken together, these findings suggest a potential association between mind–body exercise and favorable skeletal structural outcomes, whereas evidence for consistent changes in biochemical markers of bone metabolism remains limited.

### 4.1. Effects on Bone Structural Outcomes

The present meta-analysis suggests that mind–body exercise interventions, including Tai Chi, Tai Chi Rouli Ball, and Hatha yoga, may have a modest but generally positive association with bone structural outcomes in perimenopausal women. However, the overall certainty of evidence is low to very low, and these findings should therefore be interpreted cautiously [20]. For BMD, exploratory subgroup analysis suggested a between-subgroup difference across skeletal regions, whereas no significant difference was detected across exercise modalities. However, because several skeletal-region subgroups were informed by only a small number of independent studies, these apparent site-specific differences should be regarded as hypothesis-generating rather than as evidence of established differential skeletal responsiveness.

Across the included studies, Tai Chi-based interventions showed a relatively more consistent pattern at the lumbar spine, with some trials reporting improvements after prolonged practice, whereas Tai Chi Rouli Ball studies suggested potential benefits for trunk and spinal BMC [10,41,43]. A Hatha yoga trial reported improvements in whole-body and ankle BMD, although changes at axial skeletal sites were less consistent [39]. Taken together, these findings raise the possibility that differences in movement characteristics, loading distribution, and postural demands may contribute to site-specific skeletal responses [45]. However, given the small number of studies available for individual modalities and skeletal regions, these patterns remain preliminary and should be regarded as exploratory and hypothesis-generating rather than evidence of differential efficacy.

Overall, the available evidence suggests possible site-specific patterns of skeletal response; however, given the low certainty of evidence and methodological heterogeneity, these findings remain suggestive rather than definitive. Mind–body exercise may therefore be considered a supportive strategy for bone health rather than a primary osteogenic intervention [46].

### 4.2. Effects on Bone Turnover Markers and Mineral Metabolism

During the perimenopausal transition, declining ovarian function is associated with a high-turnover state of bone loss characterized by an imbalance between bone resorption and formation [47]. BTMs reflecting dynamic osteoblastic and osteoclastic activity may provide early indications of metabolic changes preceding measurable alterations in BMD.

Across the included studies (very low to low certainty of evidence), mind–body exercise interventions appear to have a modest and inconsistent influence on bone turnover. With respect to bone formation markers, findings were generally heterogeneous. For example, ALP increases of approximately 5 IU/L were reported in individual Tai Chi ball trials, whereas other comparable studies found no significant change [40]. Similarly, markers such as PINP and BGP demonstrated no consistent between-group differences, suggesting that current evidence does not clearly indicate a stable stimulation of systemic bone formation [40,44]. In terms of bone resorption, a potential reduction in β-CTX has been reported in individual studies following mind–body exercise interventions, with concurrent changes in PTH reported in some studies [44,48]. However, these patterns were not consistently replicated across studies and should therefore be interpreted cautiously [49]. Overall, these findings may suggest a possible tendency toward a less catabolic bone turnover profile, but the evidence base remains limited and inconsistent.

Mineral metabolism outcomes showed similarly mixed patterns. Some studies reported small increases in serum calcium following Tai Chi-based interventions, whereas phosphorus and magnesium levels generally remained unchanged [42]. In other studies, no clear differences were observed between intervention and control groups [43]. These findings may suggest a possible tendency toward maintenance of mineral homeostasis, although the evidence is insufficient to draw firm conclusions. The substantial heterogeneity observed across studies may be attributable to differences in exercise modalities, session duration, participant characteristics, and biochemical assessment methods. In addition, BTMs are inherently sensitive to biological variability, including circadian rhythm, fasting status, and analytical variation, which may further contribute to inconsistency across findings [50]. Therefore, the observed heterogeneity is likely multifactorial and should not be interpreted as evidence of differential intervention efficacy.

Given the very low to low certainty of evidence, these findings should be interpreted with caution. Overall, current evidence suggests that mind–body exercise is associated with small and inconsistent changes in bone turnover markers in perimenopausal women; however, the magnitude, direction, and clinical relevance of these effects remain uncertain and require confirmation from higher-quality studies. Importantly, the present review did not directly evaluate mechanistic outcomes related to bone remodeling, such as cellular signaling, mechanotransduction pathways, or tissue-level remodeling processes. Therefore, the proposed explanations involving mechanical loading, movement-specific loading patterns, or regulation of bone metabolism should be regarded as biologically plausible hypotheses rather than mechanisms demonstrated by the current evidence.

### 4.3. Practical Applications

Based on the synthesized evidence, mind–body exercises such as Tai Chi, yoga, and related modalities should be positioned as complementary, non-pharmacological lifestyle strategies rather than primary anabolic therapies for bone health. Their low-impact and accessible nature may make them suitable supportive options for perimenopausal women, although the current evidence does not establish a strong osteogenic effect [16].

Early initiation of appropriate mind–body exercise during the perimenopausal transition may be reasonable as a preventive strategy before substantial skeletal deterioration occurs. However, the available studies did not directly compare different intervention starting points across perimenopausal stages; therefore, direct evidence regarding the optimal timing of exercise initiation remains limited. Moreover, because the available evidence is more consistent with a possible bone-preserving rather than a strongly osteogenic effect, long-term adherence may be important for sustaining any potential skeletal benefits.

Current evidence does not yet support recommending particular mind–body modalities for specific skeletal regions because the observed site-specific patterns remain exploratory. Exercise selection should instead take into account feasibility, safety, adherence, and broader musculoskeletal benefits. Beyond their potential skeletal effects, mind–body exercises may also improve balance and neuromuscular function relevant to fall prevention [51].

### 4.4. Limitations

Several limitations must be acknowledged when interpreting the findings of this meta-analysis. A primary limitation concerns the overall certainty of evidence, which, assessed via the GRADE framework, ranged from very low to low across the outcomes. This degradation in evidence quality was partly attributable to methodological limitations inherent in exercise interventions. Because blinding participants to exercise allocation is generally infeasible, awareness of group assignment may influence participants’ expectations, adherence, engagement in additional physical activity, or other co-interventions, thereby increasing the risk of bias arising from deviations from the intended interventions. Nevertheless, most outcomes examined in this review, including BMD, BMC, and biochemical bone-related indicators, were objectively measured, which may reduce the direct influence of participant awareness on outcome assessment. However, incomplete reporting of whether outcome assessors or laboratory personnel were blinded means that related measurement bias cannot be completely excluded. Additionally, small sample sizes and unclear allocation concealment in several primary trials further diminish the confidence in the synthesized effect sizes.

Beyond the fundamental evidence quality, the quantitative synthesis was constrained by a relatively small pool of included studies, with only seven randomized controlled trials available for analysis. Within this limited sample, there was notable clinical heterogeneity regarding exercise modalities, frequencies, and durations. More importantly, related to the specific research theme, the perimenopausal transition is essentially characterized by substantial fluctuations in endogenous estrogen. Definitions and staging approaches for perimenopause also varied considerably across the included trials. Some studies used explicit menstrual-history, endocrine, or STRAW-based criteria, whereas others relied primarily on age, menopausal symptoms, or broader author-defined classifications without standardized reproductive staging. Consequently, participants classified as perimenopausal may have represented different physiological stages of the menopausal transition. This variability in reproductive-stage ascertainment may have contributed to clinical heterogeneity and restricted our ability to explore menopausal stage as a potential source of statistical heterogeneity through more detailed subgroup analyses.

The limited number of independent studies constrained the precision and reliability of several subgroup analyses, particularly because some subgroup categories were represented by only one or two studies. Accordingly, between-subgroup differences could not be characterized reliably, and these findings should be regarded as exploratory and hypothesis-generating rather than confirmatory. The small number of independent study clusters also limits the strength of inference from the CR2-adjusted analyses. Although CR2 small-sample correction was applied, these analyses were based on only six independent studies for BMD, three for BMC and BMM, and four for BTMs, with low effective degrees of freedom for some outcomes. Therefore, the CR2 analyses should be regarded as supplementary checks rather than independent confirmation of robustness. The limited evidence base also affected analyses of biochemical outcomes, for which pooled estimates were more susceptible to the influence of individual trials and did not support comprehensive meta-regression. Differences in DXA measurement protocols may additionally have contributed to heterogeneity in BMD and BMC estimates by reducing comparability across studies. Publication bias also remains a potential concern. Given that only three to six independent studies contributed to the outcome-specific analyses and multiple effect sizes within studies were statistically dependent, the funnel plots and Egger’s regression tests have limited interpretability. Thus, these assessments should be considered exploratory, and non-significant findings cannot exclude publication bias or small-study effects.

Another methodological limitation concerns the estimation of change-score standard deviations. Because the primary trials did not report pre–post Pearson correlation coefficients (r), we assumed r = 0.5 in accordance with Cochrane recommendations. Although sensitivity analyses using r values from 0.3 to 0.7 yielded broadly similar estimates for structural outcomes, assuming a common correlation across studies and outcomes introduces additional uncertainty. Overall, the quantitative findings should be interpreted cautiously and require confirmation in larger, rigorously designed randomized trials with improved reporting of adherence and co-interventions and blinded outcome assessment where feasible.

## 5. Conclusions

In conclusion, this systematic review and meta-analysis found that mind–body exercise was associated with favorable pooled estimates for skeletal structural outcomes, particularly BMD and BMC, in perimenopausal women. However, the magnitude and consistency of these associations remain uncertain because of substantial heterogeneity, prediction intervals crossing zero for some outcomes, and the low to very low certainty of the available evidence. Given this limited certainty, the current findings should not be interpreted as supporting definitive clinical recommendations for the use of mind–body exercise to improve bone health in perimenopausal women. No distinct or consistent alterations were observed in biochemical markers of bone turnover or systemic mineral metabolism. At present, mind–body modalities may be considered supportive components of healthy aging and bone health management rather than established osteogenic interventions. Their potential role in preserving bone health should therefore be interpreted cautiously and confirmed in higher-quality studies. To meaningfully advance this field, subsequent larger, rigorously designed randomized controlled trials should move beyond general participant classifications by incorporating standardized biological sampling, standardized reproductive and endocrine staging, and tight control of confounding lifestyle factors. Future studies should also systematically evaluate exercise dose–response relationships across intervention intensity, frequency, session duration, and overall intervention period and include sufficiently long follow-up to assess clinically meaningful skeletal outcomes, particularly fragility fractures. Such evidence may help clarify which intervention characteristics are most relevant across different stages of the menopausal transition and inform the development of more evidence-based and tailored exercise recommendations for menopausal health.

## Figures and Tables

**Table 1:** Study eligibility criteria.

	Inclusion	Exclusion
P	Participants were perimenopausal women, as defined in the original studies; studies with mixed populations were included only when data for perimenopausal women could be extracted separately.	Studies were excluded if participants were not perimenopausal women or relevant data could not be extracted separately.
I	The intervention consisted of a single mind–body exercise modality, such as Tai Chi, Baduanjin, Qigong, yoga, Pilates, Wuqinxi, or Yijinjing, without restrictions on exercise frequency, session duration, or intervention period.	The intervention was not a mind–body exercise; mind–body exercise was combined with medication, nutritional supplementation, other exercise modalities, physical therapy, or additional interventions
C	Eligible comparators included no intervention, a wait-list control, usual lifestyle, usual care, health education, or routine daily activities.	Studies without control groups or with high-intensity exercise as control
O	Studies reported at least one bone health outcome, including bone mineral density (BMD), bone mineral content (BMC), bone mineral metabolism (BMM; serum calcium, magnesium, and phosphorus), or bone turnover markers (BTMs), with sufficient data to calculate an effect size.	No bone health-related outcomes
S	Randomized controlled trials or other controlled clinical intervention studies reported as full-text journal articles, theses, or dissertations were eligible.	Qualitative studies, systematic reviews, meta-analyses, study protocols, conference abstracts, or reports without sufficient methodological or outcome data.

Note: P, participants; I, intervention; C, control; O, outcome; S, study design.

**Table 2:** Subgroup analyses based on meta-analysis results of BMD.

Subgroup	Studies	K	N	Hedges’ g	95% CI	T-Value	P_effect_	I^2^-2	I^2^-3	P_between_
Body Part										**0.0273**
Hip	4	16	193	0.42	[0.11, 0.74]	2.7193	**0.0106**	29.08%	0%	
Lower limb	3	3	112	0.75	[0.18, 1.32]	2.6937	**0.0113**	25.08%	25.08%	
Spine	4	8	193	0.65	[0.28, 1.01]	3.5945	**0.0011**	0%	24.54%	
Trunk	2	5	82	0.91	[0.42, 1.41]	3.7417	**0.0007**	70.19%	0%	
Upper limb	2	2	82	0.47	[−0.20, 1.14]	1.4186	0.1660	0%	0%	
Whole body	3	3	82	0.13	[−0.37, 0.62]	0.5311	0.5991	34.90%	34.90%	
Session Duration										0.5641
90 min	2	12	70	0.66	[0.18, 1.14]	2.8119	**0.0080**	30.25%	0%	
60 min	4	25	205	0.50	[0.20, 0.80]	3.3965	**0.0017**	55.19%	0%	
Frequency										0.7788
4.5 times/week	1	12	30	0.53	[0.26, 0.81]	3.9916	**0.0003**	0%	0%	
3 times/week	5	3	245	0.61	[−0.08, 1.36]	1.8015	0.0802	55.99%	0%	
Mind–body Type										0.6533
Tai Chi chuan	2	16	123	0.46	[0.06, 0.83]	2.3332	**0.0257**	0%	15.37%	
Hatha yoga	1	3	30	0.85	[0.03, 1.67]	2.0963	**0.0436**	38.99%	0%	
Tai Chi Rouli Ball	3	18	122	0.61	[0.21, 0.95]	3.1769	**0.0032**	64.39%	0%	

Note: Subgroup analyses were conducted based on meta-analysis results for BMD. Studies denotes the number of independent studies; K denotes the number of effect sizes; N denotes the number of unique participants (counted only once when the same participants contributed multiple effect sizes); Hedges’ g represents the standardized mean difference; CI indicates confidence interval; P_effect_ refers to the *p*-value for the pooled effect within each subgroup; P_between_ denotes the *p*-value for between-group comparisons; I^2^-2 and I^2^-3 represent within-study and between-study heterogeneity, respectively. Statistically significant results (*p* < 0.05) are highlighted in bold. Given that several subgroup categories were informed by only one or two independent studies, all subgroup findings should be interpreted cautiously and regarded as exploratory and hypothesis-generating rather than confirmatory.

**Table 3:** Subgroup analyses based on meta-analysis results of BMC.

Subgroup	Studies	K	N	Hedges’ g	95% CI	T-Value	P_effect_	I^2^-2	I^2^-3	P_between_
Body Part										0.7248
Hip	1	3	40	2.81	[0.39, 5.25]	2.5246	**0.0267**	97.35%	0%	
Lower limb	2	2	82	0.81	[−1.69, 3.31]	0.7045	0.4945	0%	0%	
Spine	3	5	122	1.70	[−0.10, 3.50]	2.0548	0.0623	0%	94.75%	
Trunk	2	4	82	1.30	[−0.66, 3.26]	1.4493	0.1729	0%	0%	
Upper limb	2	2	82	2.24	[−0.29, 4.77]	1.9282	0.0788	0%	0%	
Whole body	2	2	82	0.82	[−1.67, 3.32]	0.7187	0.4861	0%	0%	
Session Duration										0.5552
90 min	2	12	70	1.47	[0.62, 3.24]	3.1241	**0.0065**	91.08%	0%	
60 min	1	6	52	1.93	[0.50, 2.43]	3.2227	**0.0053**	87.81%	0%	
Frequency										0.7932
4.5 times/week	1	6	30	1.78	[0.38, 3.18]	2.6947	**0.0159**	50.63%	0%	
3 times/week	2	12	92	1.57	[0.62, 2.51]	3.5137	**0.0029**	93.85%	0%	

Note: Subgroup analyses were conducted based on meta-analysis results for BMC. Studies denotes the number of independent studies; K denotes the number of effect sizes; N denotes the number of unique participants (counted only once when the same participants contributed multiple effect sizes); Hedges’ g represents the standardized mean difference; CI indicates confidence interval; P_effect_ refers to the *p*-value for the pooled effect within each subgroup; P_between_ denotes the *p*-value for between-group comparisons; I^2^-2 and I^2^-3 represent within-study and between-study heterogeneity, respectively. Statistically significant results (*p* < 0.05) are highlighted in bold. Given that several subgroup categories were informed by only one or two independent studies, all subgroup findings should be interpreted cautiously and regarded as exploratory and hypothesis-generating rather than confirmatory.

**Table 4:** Subgroup analyses based on meta-analysis results of BMM.

Subgroup	Studies	K	N	Hedges’ g	95% CI	T-Value	P_effect_	I^2^-2	I^2^-3	P_between_
Mineral Type										**0.0031**
Ca	3	3	160	1.40	[0.21, 2.58]	3.0348	**0.0289**	42.09%	42.09%	
Mg	2	2	82	0.85	[−0.36, 2.07]	1.8048	0.1309	18.81%	18.81%	
P	3	3	160	0.29	[−0.87, 1.46]	0.6478	0.5457	42.19%	42.19%	
Frequency										0.6956
4.5 times/week	1	3	30	1.23	[−1.22, 3.67]	1.23	0.2656	72.59%	0%	
3 times/week	2	5	130	0.73	[−0.97, 2.43]	1.0440	0.3367	25.17%	68.14%	
Mind–Body Type										0.0651
Tai Chi Rouli Ball	2	6	82	1.3240	[0.52, 2.13]	4.0164	**0.0070**	60.67%	0%	
Wu Xing Jian Gu Cao	1	2	78	0.0160	[−1.15, 1.18]	0.0334	0.9744	90.57%	0%	
Session Duration										0.2511
90 min	1	3	30	1.23	[−0.25, 2.70]	2.1390	0.0854	72.59%	0%	
60 min	1	3	52	1.41	[0.05, 2.77]	2.6641	**0.0447**	64.15%	0%	
37.5 min	1	2	78	0.02	[−1.44, 1.47]	0.0282	0.9786	90.57%	0%	

Note: Subgroup analyses were conducted based on meta-analysis results for BMM. Studies denotes the number of independent studies; K denotes the number of effect sizes; N denotes the number of unique participants (counted only once when the same participants contributed multiple effect sizes); Hedges’ g represents the standardized mean difference; CI indicates confidence interval; P_effect_ refers to the *p*-value for the pooled effect within each subgroup; P_between_ denotes the *p*-value for between-group comparisons; I^2^-2 and I^2^-3 represent within-study and between-study heterogeneity, respectively. Statistically significant results (*p* < 0.05) are highlighted in bold. Given that several subgroup categories were informed by only one or two independent studies, all subgroup findings should be interpreted cautiously and regarded as exploratory and hypothesis-generating rather than confirmatory.

**Table 5:** Subgroup analyses based on meta-analysis results of BTMs.

Subgroup	Studies	K	N	Hedges’ g	95% CI	T-Value	P_effect_	I^2^-2	I^2^-3	P_between_
Marker Type										0.1990
Bone formation markers	4	6	122	−0.03	[−0.47, 0.42]	−0.1549	0.8829	1.26%	0%	
Bone resorption markers	1	1	78	−0.54	[−1.42, 0.33]	−1.5917	0.1723	na	na	
Frequency										0.8910
4.5 times/week	1	1	30	−0.17	[−1.43, 1.10]	−0.3353	0.7510	na	na	
3 times/week	3	6	170	−0.09	[−0.59, 0.41]	−0.4534	0.6693	31.83%	22.97%	
Mind–Body Type										0.3589
Tai Chi Rouli Ball	3	4	122	0.06	[−0.55, 0.66]	0.2483	0.8138	39.45%	0%	
Wu Xing Jian Gu Cao	1	3	78	−0.30	[−0.98, 0.38]	−1.3330	0.3086	20.47%	0%	
Session Duration										0.6415
90 min	2	3	70	0.10	[−0.73, 0.94]	0.3420	0.7496	57.89%	0%	
60 min	1	1	52	−0.03	[−1.25, 1.19]	−0.0651	0.9512	na	na	
37.5 min	1	3	78	−0.30	[−1.07, 0.47]	−1.0760	0.3425	20.47%	0%	

Note: Subgroup analyses were conducted based on meta-analysis results for BTMs. Studies denotes the number of independent studies; K denotes the number of effect sizes; N denotes the number of unique participants (counted only once when the same participants contributed multiple effect sizes); Hedges’ g represents the standardized mean difference; CI indicates confidence interval; P_effect_ refers to the *p*-value for the pooled effect within each subgroup; P_between_ denotes the *p*-value for between-group comparisons; I^2^-2 and I^2^-3 represent within-study and between-study heterogeneity, respectively. na indicates not applicable because heterogeneity could not be estimated when only one effect size was available. Given that several subgroup categories were informed by only one or two independent studies, all subgroup findings should be interpreted cautiously and regarded as exploratory and hypothesis-generating rather than confirmatory.

**Table 6:** Sensitivity analysis for assumed pre-post Pearson’s correlation coefficients.

Variables	r	SMD	95% CI	T-Value	*p*
	0.3	0.47	[0.23, 0.71]	3.9916	0.0003
Bone Mineral Density	0.5	0.55	[0.29, 0.80]	4.3744	<0.0001
	0.7	0.69	[0.41, 0.97]	4.9676	<0.0001
	0.3	1.28	[0.68, 1.88]	4.5258	0.0003
Bone Mineral Content	0.5	1.63	[0.86, 2.40]	4.4810	0.0003
	0.7	1.85	[0.99, 2.72]	4.5340	0.0003
	0.3	0.76	[−0.14, 1.66]	2.0009	0.0855
Bone Mineral Metabolism	0.5	0.90	[−0.14, 1.92]	2.0542	0.0790
	0.7	1.14	[−0.13, 2.40]	2.1271	0.0710
	0.3	−0.09	[−0.48, 0.30]	−0.55	0.6029
Bone Turnover Markers	0.5	−0.10	[−0.53, 0.33]	−0.57	0.5863
	0.7	−0.12	[−0.61, 0.38]	−0.57	0.5867

Note: Sensitivity analysis for assumed pre–post Pearson’s correlation coefficients. r denotes the assumed pre–post Pearson’s correlation coefficient; SMD, standardized mean difference; 95% CI.lb and 95% CI.ub represent the lower and upper bounds of the 95% confidence interval, respectively.

**Table 7:** GRADE assessment results.

Outcome	No. of Participants (Studies)	Certainty Assessment						Effect Size (SMD [95% CI])	Certainty (GRADE)
		Risk of Bias	Inconsistency	Indirectness	Imprecision	Publication Bias	Other		
Bone Mineral Density	275 (6)	Serious	Serious	Not serious	Not serious	Serious	None	0.55 [0.29, 0.80]	Very Low
Bone Mineral Content	122 (3)	Serious	Very Serious	Not serious	Not serious	Serious	None	1.63 [0.86, 2.40]	Very Low
Bone Mineral Metabolism	160 (3)	Serious	Very Serious	Not serious	Serious	Serious	None	0.90 [−0.14, 1.92]	Very Low
Bone Turnover Markers	200 (4)	Serious	Serious	Not serious	Serious	Serious	None	−0.10 [−0.53, 0.33]	Very Low

Note: The certainty of evidence according to the Grading of Recommendations, Assessment, Development, and Evaluations (GRADE) system is categorized into four levels: high, moderate, low, and very low. High certainty means we are very confident in the estimated effect, while moderate certainty indicates moderate confidence. Low certainty reflects limited confidence in the estimated effect and very low certainty indicates very little confidence. This classification aids in assessing the reliability of research findings and guides the interpretation and application of evidence. In this review, publication bias was rated as “Serious” across all outcomes; although a comprehensive search strategy was employed, the limited number of included independent studies (k < 10) precluded reliable statistical or visual detection of small-study effects, meaning undetected publication bias could not be definitively ruled out.

**Figure A1:**
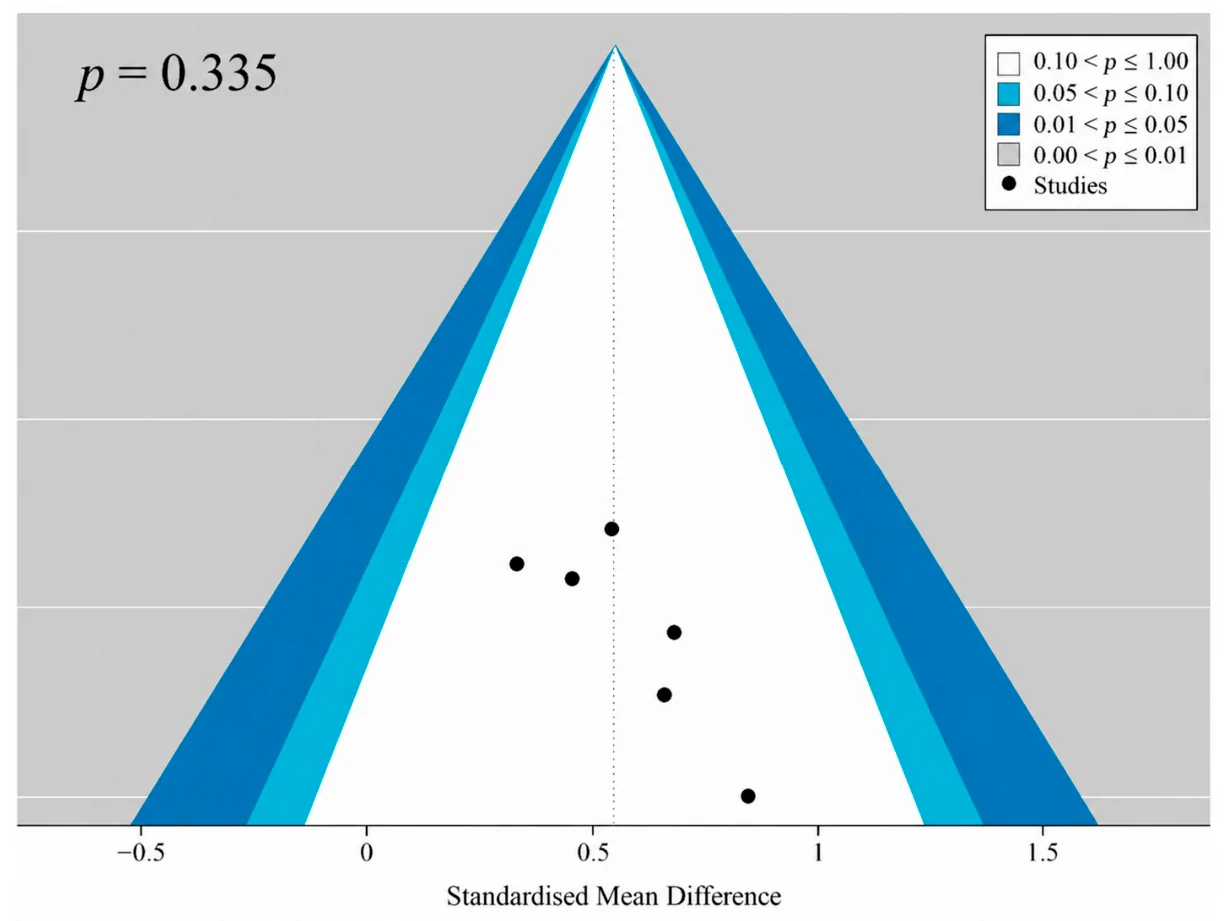
BMD funnel plot.

**Figure A2:**
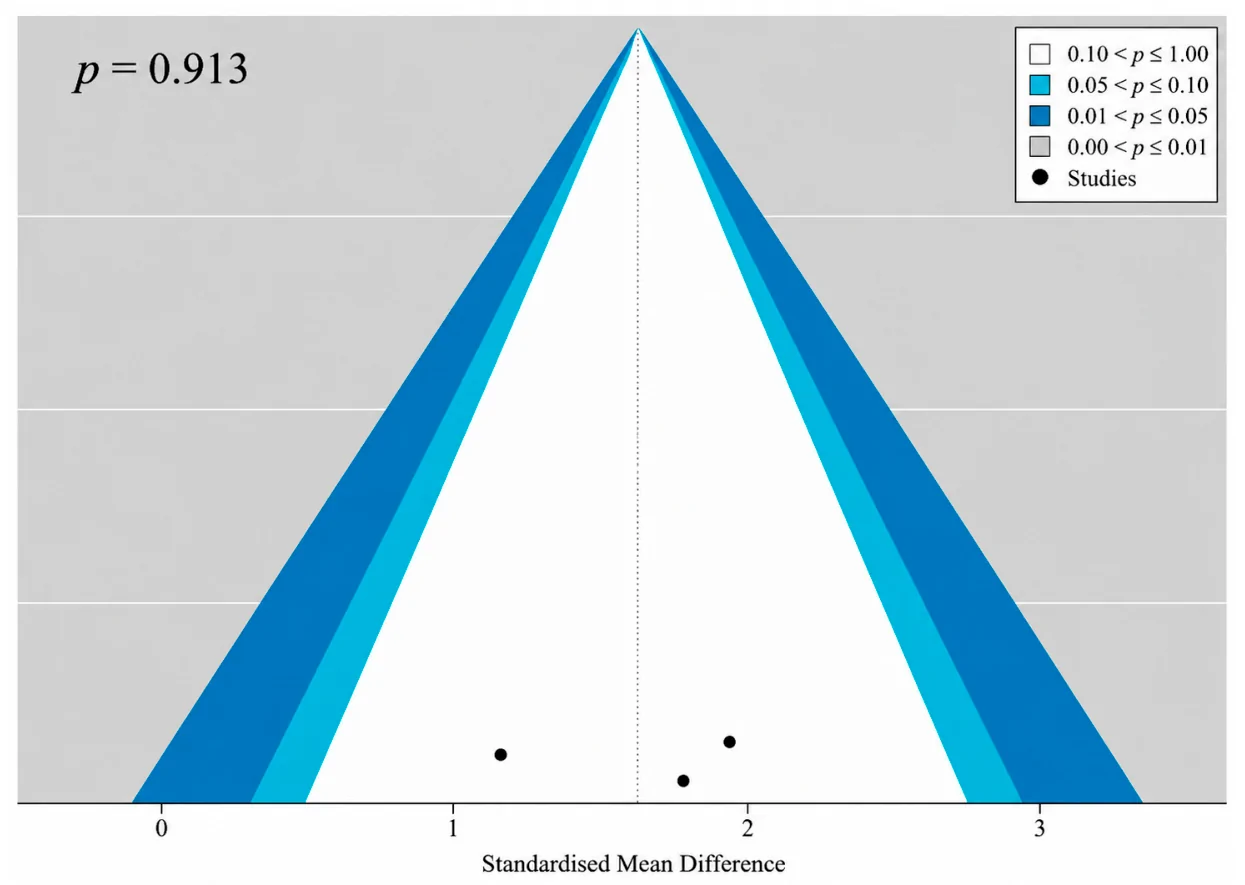
BMC funnel plot.

**Figure A3:**
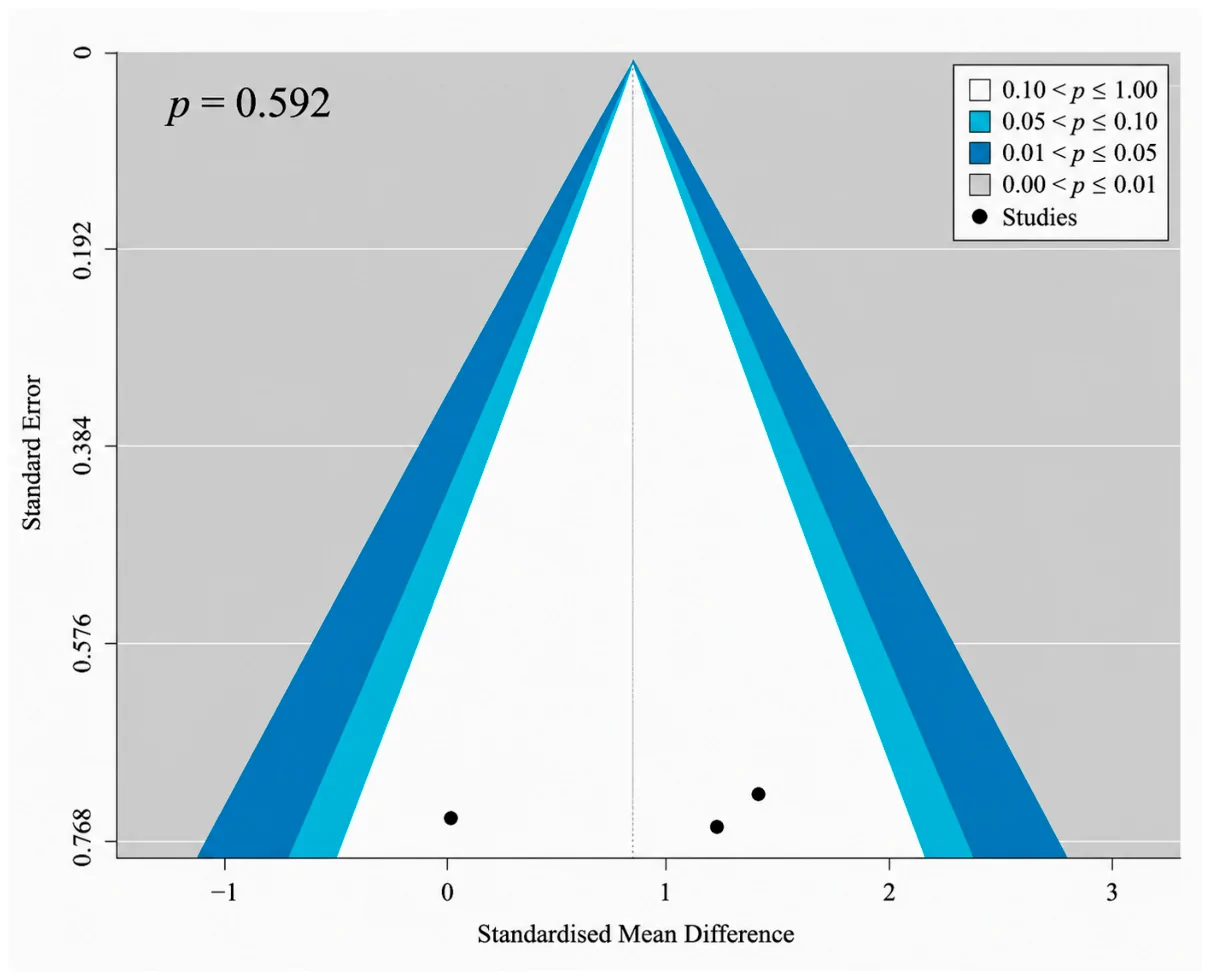
BMM funnel plot.

**Figure A4:**
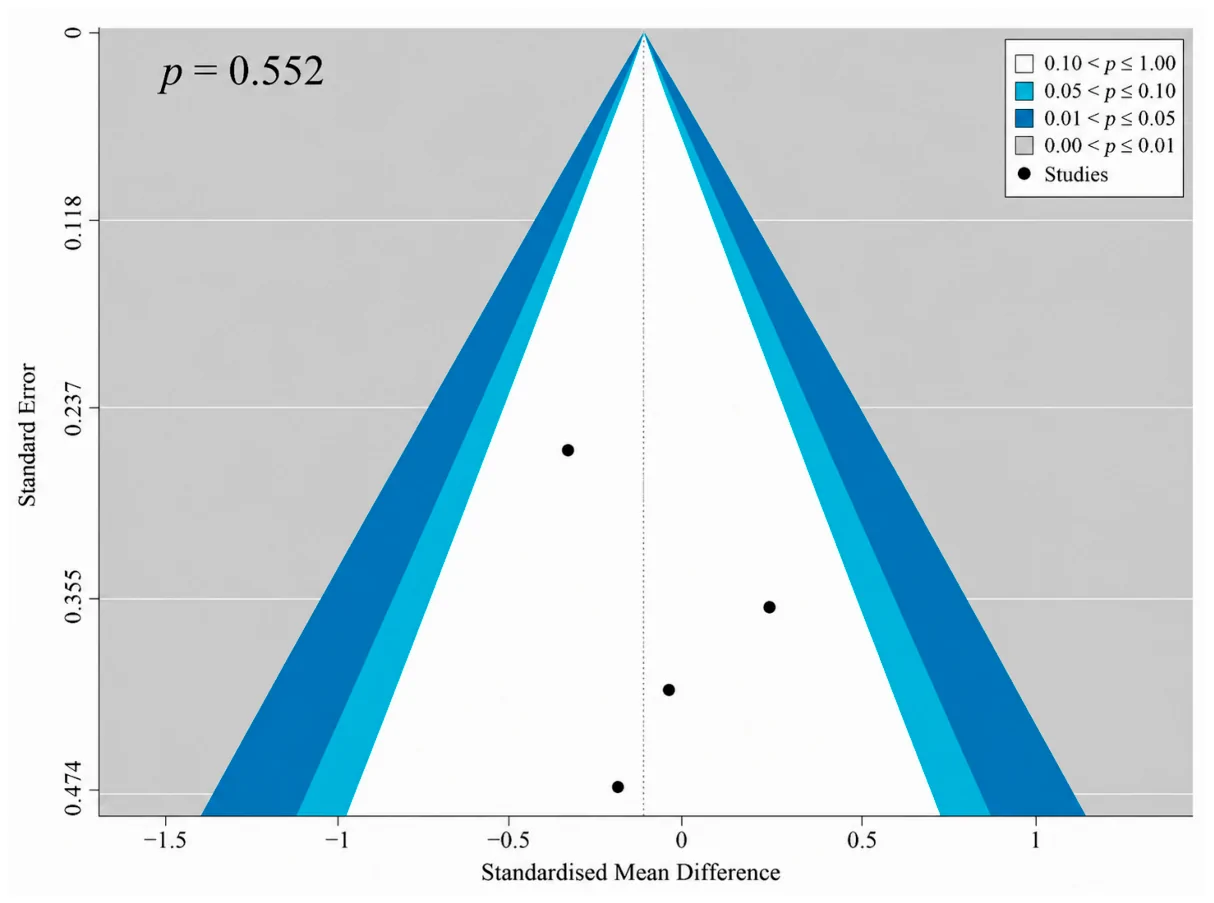
BTMs funnel plot.

**Figure A5:**
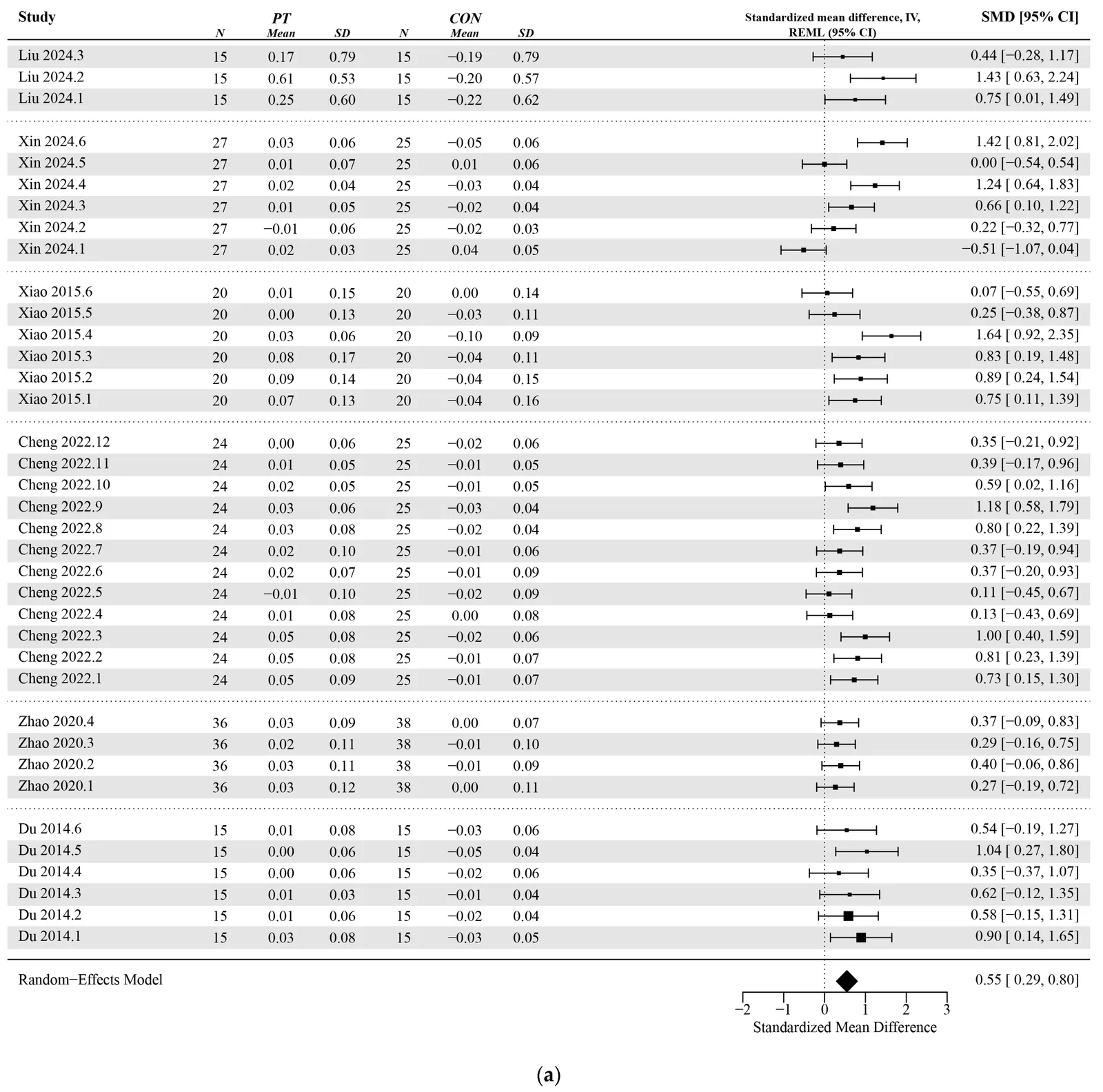

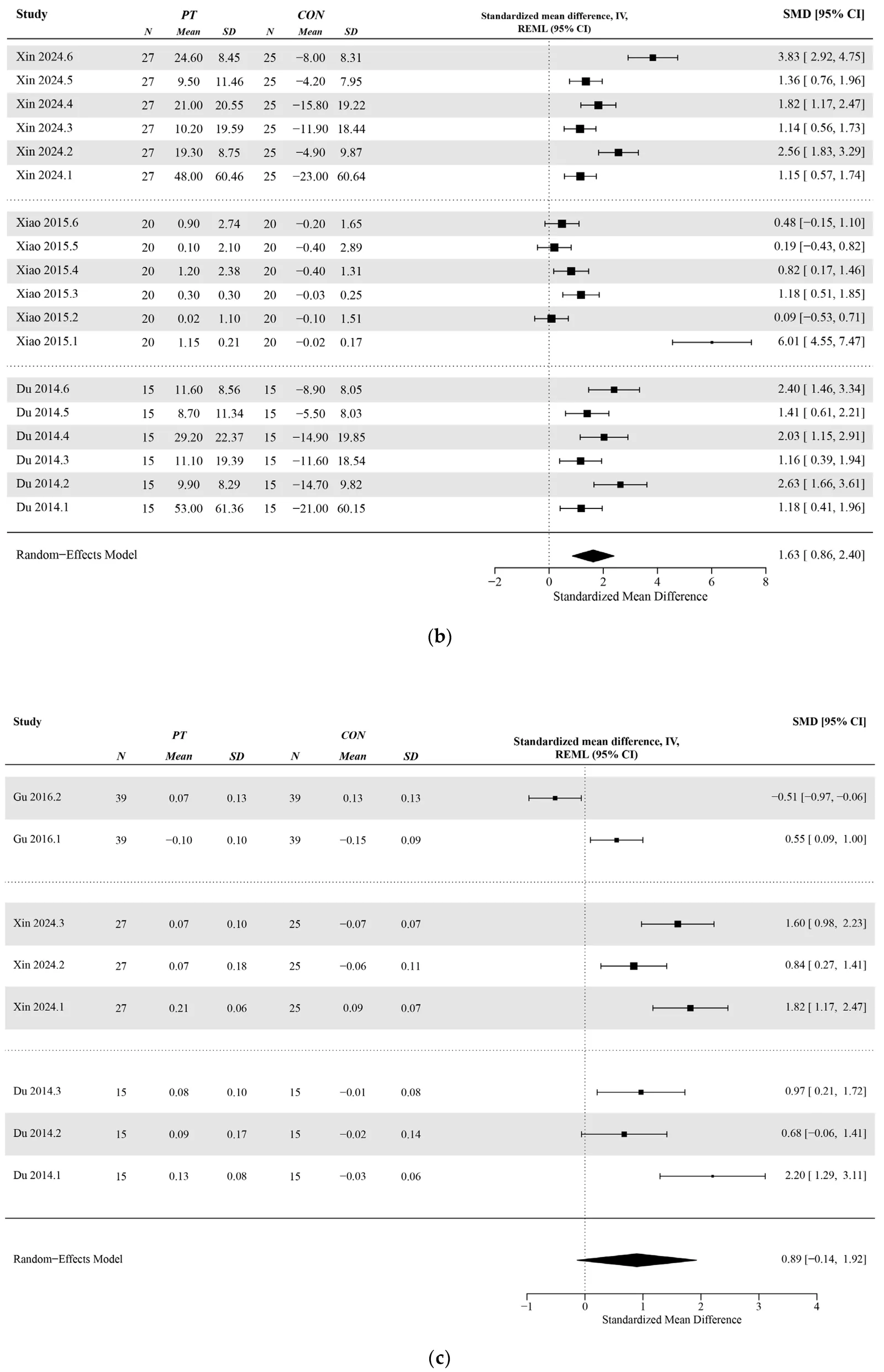

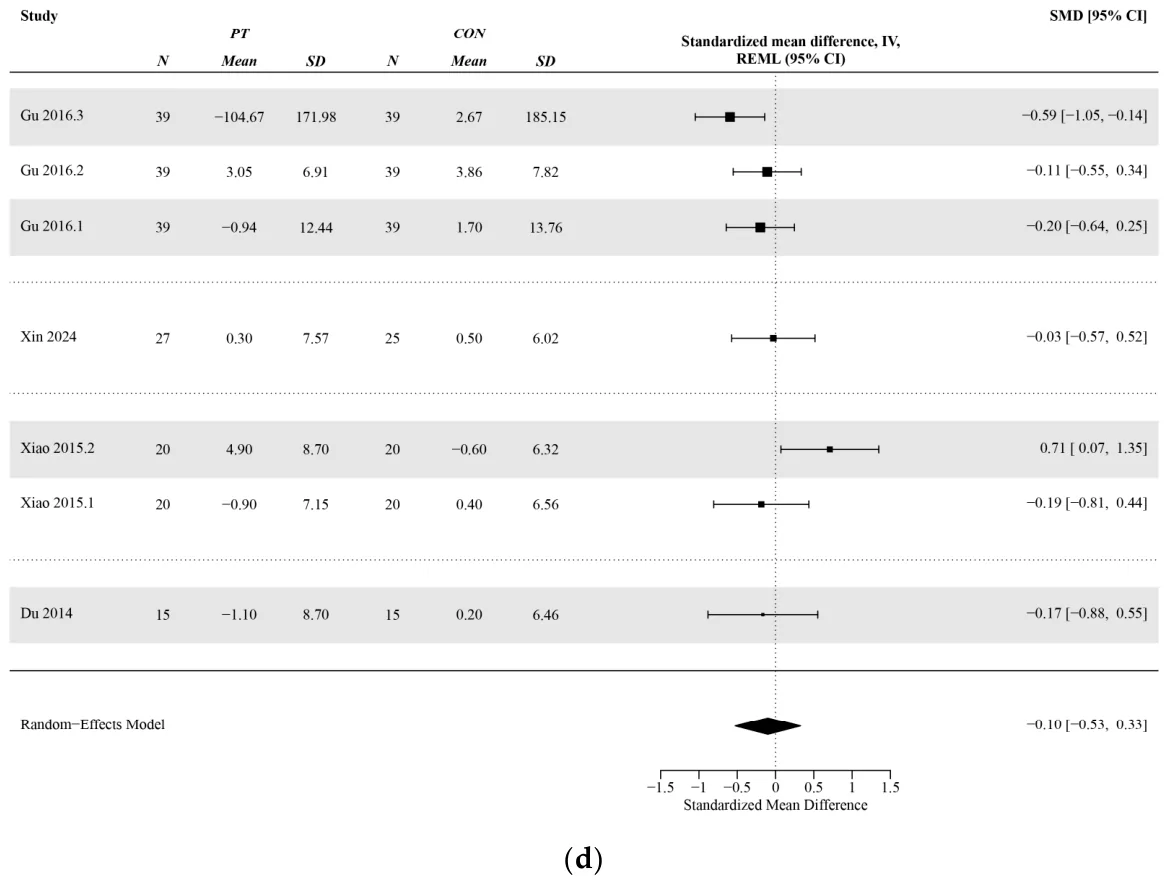
(**a**) BMD forest plot [10,39,40,41,42,43]. (**b**) BMC forest plot [40,42,43]. (**c**) BMM forest plot [42,43,44]. (**d**) BTMs forest plot [40,42,43,44].

**Figure A6:**
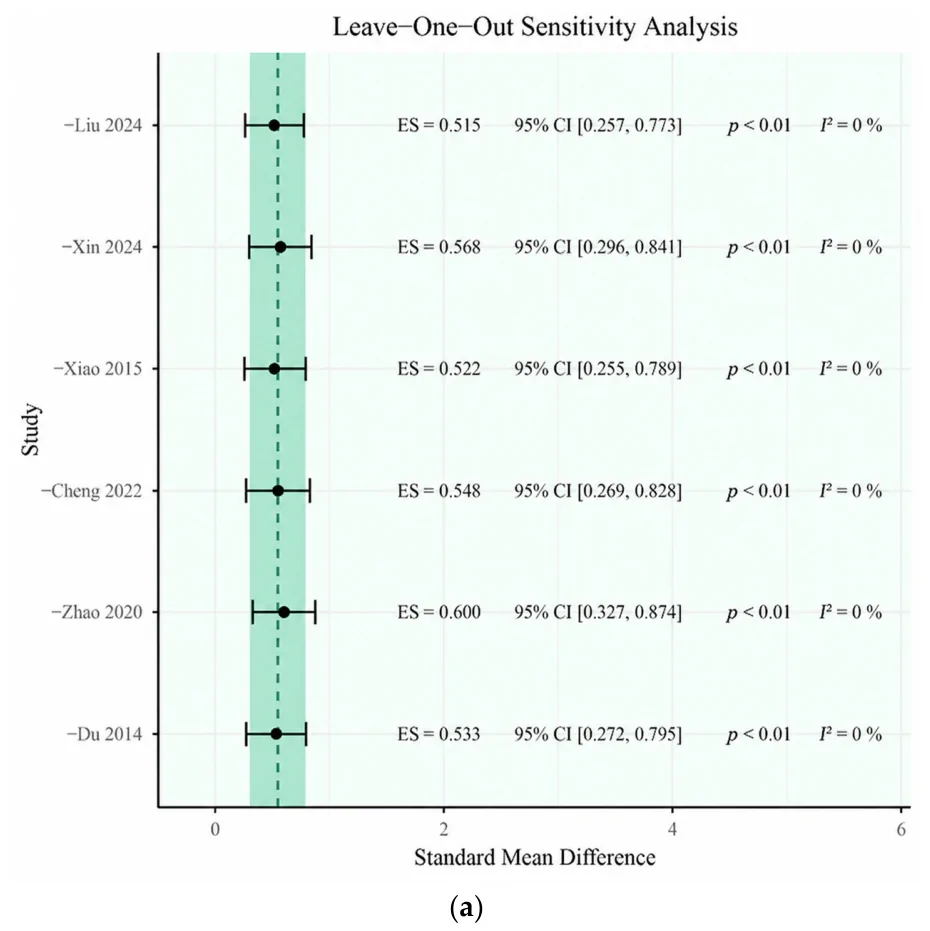

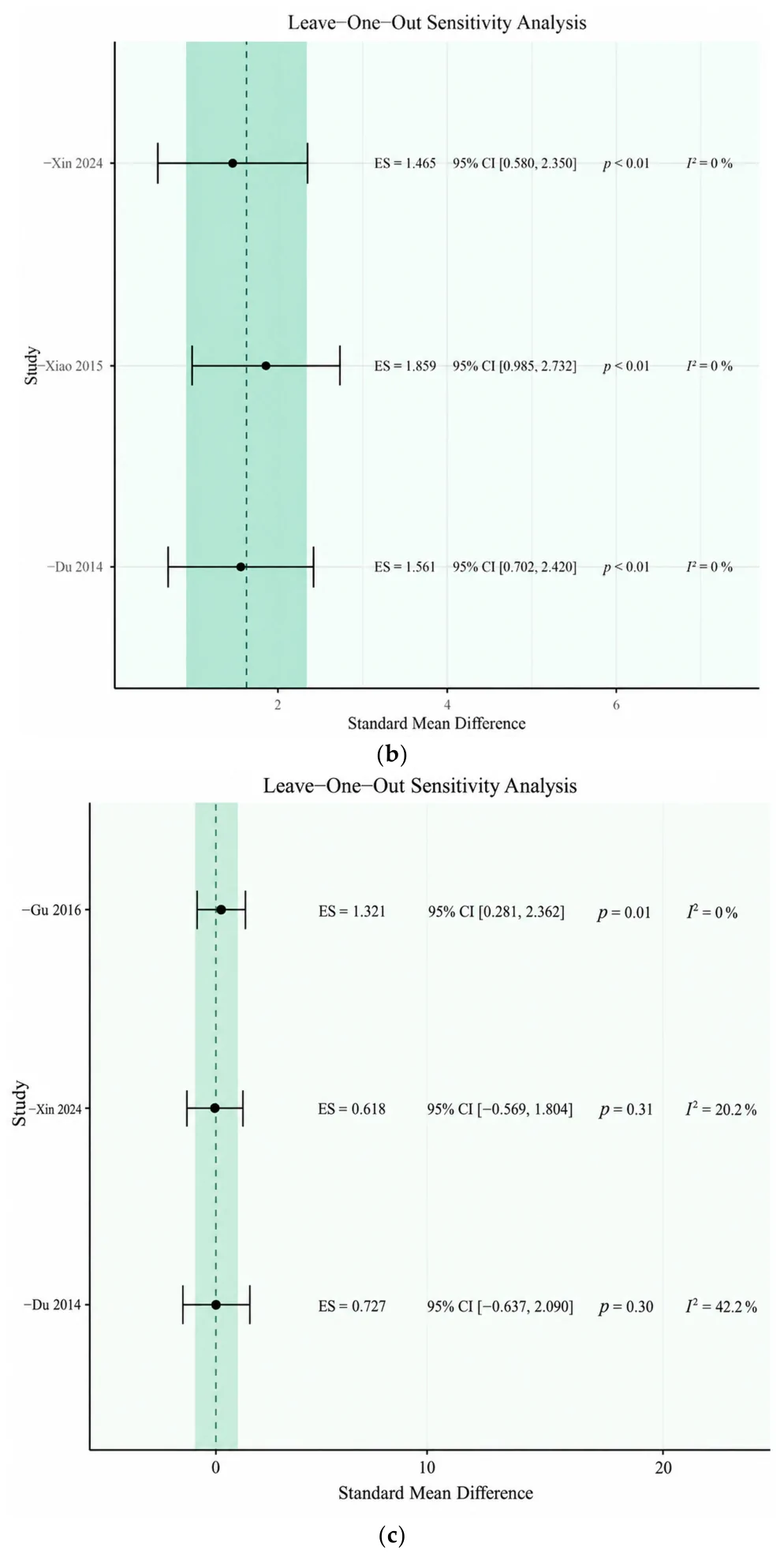

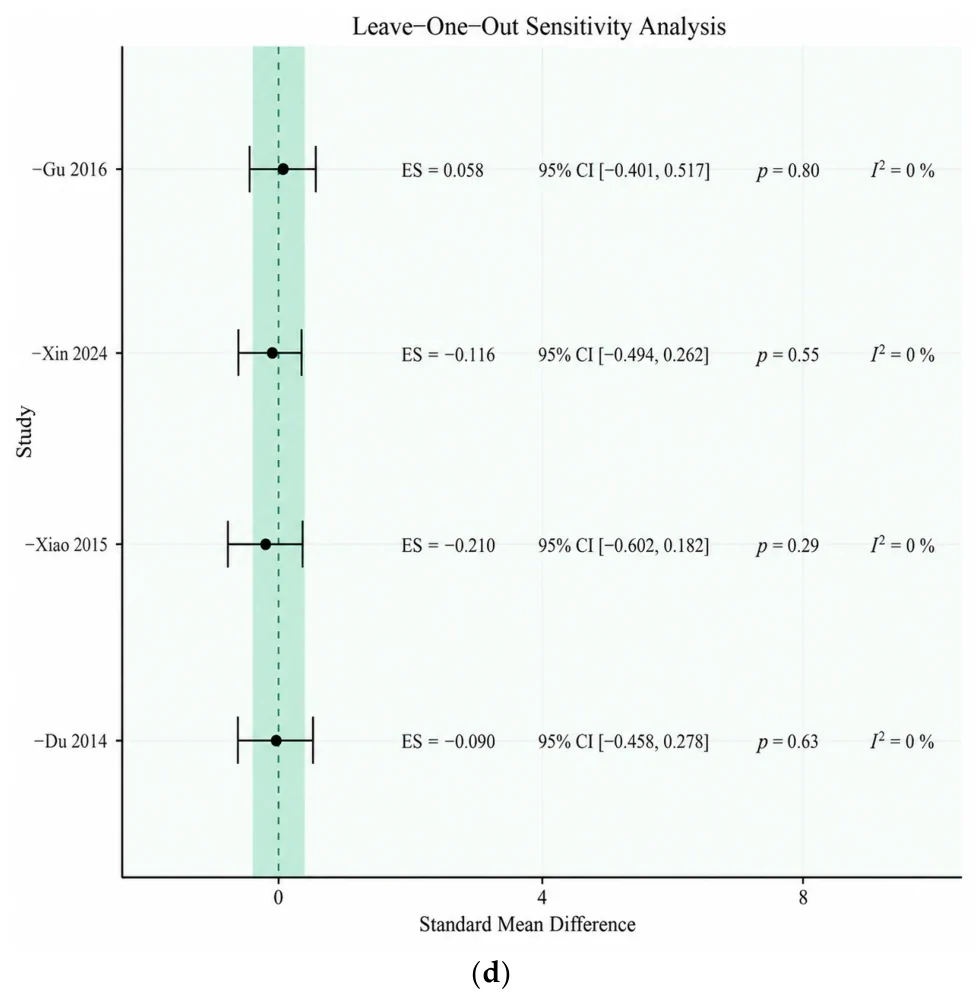
(**a**) BMD leave-one-out sensitivity analysis plot [10,39,40,41,42,43]. (**b**) BMC leave-one-out sensitivity analysis plot [40,42,43]. (**c**) BMM leave-one-out sensitivity analysis plot [42,43,44]. (**d**) BTMs leave-one-out sensitivity analysis plot [40,42,43,44].

## Data Availability

The corresponding author of this article will unconditionally provide all the original data supporting the results of this study.

## Supplementary Materials

The following supporting information can be downloaded at: https://www.mdpi.com/article/10.3390/life16091389/s1, Supplementary Materials Table S1: Prisma Checklist 2020 [19]; Supplementary Materials Table S2: Rob2 judgement reason [10,39,40,41,42,43,44].

## Appendix A

**Table A1:** The Detailed Search Strategy.

Data	Query	Results	Updated
PubMed	((“Menopause”[Mesh] OR “Perimenopause”[Mesh] OR “Postmenopause”[Mesh] OR menopaus * [Title/Abstract] OR perimenopause * [Title/Abstract] OR postmenopaus * [Title/Abstract] OR climacteric * [Title/Abstract]) AND (“Mind-Body Therapies” [Mesh] OR “Tai Ji” [Mesh] OR “Yoga” [Mesh] OR “Qigong” [Mesh] OR “mind-body” [Title/Abstract] OR “meditative movement” [Title/Abstract] OR “mindful movement” [Title/Abstract] OR tai chi [Title/Abstract] OR taichi [Title/Abstract] OR taiji [Title/Abstract] OR qigong [Title/Abstract] OR yoga [Title/Abstract] OR pilates [Title/Abstract]))	532	10
Web of Science	TS = ((“Menopause” OR “Perimenopause” OR “Postmenopause” OR menopaus * OR perimenopause * OR postmenopaus * OR climacteric *) AND (“Mind-Body Therapies” OR “Tai Ji” OR “Yoga” OR “Qigong” OR “mind-body” OR “meditative movement” OR “mindful movement” OR “tai chi” OR taichi OR taiji OR qigong OR yoga OR pilates))	858	10
Scopus	TITLE-ABS-KEY((“Menopause” OR “Perimenopause” OR “Postmenopause” OR menopaus * OR perimenopause * OR postmenopaus * OR climacteric *) AND (“Mind-Body Therapies” OR “Tai Ji” OR “Yoga” OR “Qigong” OR “mind-body” OR “meditative movement” OR “mindful movement” OR “tai chi” OR taichi OR taiji OR qigong OR yoga OR pilates))	726	17
EBSCO-SPORTDiscus	TX ((“Menopause” OR “Perimenopause” OR “Postmenopause” OR menopaus * OR perimenopause * OR postmenopaus * OR climacteric *) AND (“Mind-Body Therapies” OR “Tai Ji” OR “Yoga” OR “Qigong” OR “mind-body” OR “meditative movement” OR “mindful movement” OR “tai chi” OR taichi OR taiji OR qigong OR yoga OR pilates))	1320	31
EBSCO-Medline	((MH “Menopause+” OR MH “Perimenopause+” OR MH “Postmenopause+” OR TX menopaus * OR TX perimenopause * OR TX postmenopaus * OR TX climacteric *) AND (MH “Mind-Body Therapies+” OR MH “Tai Ji+” OR MH “Yoga+” OR MH “Qigong+” OR TX “mind-body” OR TX “meditative movement” OR TX “mindful movement” OR TX “tai chi” OR TX taichi OR TX taiji OR TX qigong OR TX yoga OR TX pilates)) NOT (MH animals NOT MH humans)	1347	25
Cochrane Library	#1: ([mh “Menopause”] OR [mh “Perimenopause”] OR [mh “Postmenopause”] OR menopaus *:ti,ab,kw OR perimenopause *:ti,ab,kw OR postmenopaus *:ti,ab,kw OR climacteric *:ti,ab,kw) #2 ([mh “Mind-Body Therapies”] OR [mh “Tai Ji”] OR [mh “Yoga”] OR [mh “Qigong”] OR “mind-body”:ti,ab,kw OR “meditative movement”:ti,ab,kw OR “mindful movement”:ti,ab,kw OR “tai chi”:ti,ab,kw OR taichi:ti,ab,kw OR taiji:ti,ab,kw OR qigong:ti,ab,kw OR yoga:ti,ab,kw OR pilates:ti,ab,kw) #3: #1 AND #2	351	17
VIP	((M = (menopause OR perimenopause OR postmenopause OR climacteric) OR R = (menopause OR perimenopause OR postmenopause OR climacteric)) AND (M = (mind-body exercise OR mind-body therapy OR mind-body intervention OR meditative exercise OR mindfulness-based exercise OR Tai Chi OR Taijiquan OR Qigong OR Baduanjin OR Yijinjing OR Wuqinxi OR yoga OR Pilates) OR R = (mind-body exercise OR mind-body therapy OR mind-body intervention OR meditative exercise OR mindfulness-based exercise OR Tai Chi OR Taijiquan OR Qigong OR Baduanjin OR Yijinjing OR Wuqinxi OR yoga OR Pilates)))	423	6
WanFang	Topic = (menopause OR perimenopause OR postmenopause OR climacteric) AND Topic = (mind-body exercise OR mind-body therapy OR mind-body intervention OR Tai Chi OR Taijiquan OR Qigong OR Baduanjin OR Yijinjing OR Wuqinxi OR yoga OR Pilates OR meditative exercise OR mindfulness-based exercise)	580	19
Chinese Medical Journals Full-text	(menopause OR perimenopause OR climacteric) AND (Tai Chi OR Taijiquan OR Qigong OR Baduanjin OR yoga OR Pilates OR mind-body exercise OR mind-body therapy)	2	0

Note: ti = title; ab = abstract; kw = keywords; tw = text words; TS = topic; TX = all text; SU = subject terms; M = MeSH term. The asterisk (*) indicates a truncation symbol used to retrieve different word endings.

## Appendix B

**Table A2:** The Characteristics of the Studies Included.

First Author	Design	Participants	Terms	Definition of Perimenopausal Stage	Exp Sessions	Con Sessions	Dur (min)	Fre	Wk
Du, 2014 [43]	RCT	Exp—N: 15 Con—N: 15 Exp—Age: 51.69 ± 3.18 Con—Age: 52.21 ± 3.01	Tai Chi Rouli ball	Perimenopause (45–55 years); no specific menstrual or endocrine staging criteria were reported. The authors broadly defined perimenopause as the period from the onset of menopause-related endocrine, biological, and clinical features to 1 year after the final menstrual period.	Tai Chi Rouli ball Routine 1–3 Intensity: Heart rate 110–130 beats/min.	The control group did not exercise and maintained their original lifestyle.	90	4.5	24
Zhao, 2020 [41]	RCT	Exp—N: 36 Con—N: 38 Exp—Age: 49.7 ± 3.9 Con—Age: 49.7 ± 3.9	Tai Chi Quan	Perimenopause (45–55 years), defined by clinically diagnosed perimenopausal symptoms and a Kupperman score ≥ 16. Menstrual status was reported as menopausal, non-menopausal, or irregular, but no specific operational criteria or endocrine staging system was provided.	Under the guidance of a professional Tai Chi coach (group teaching), a 48-week, 24-style, simplified Tai Chi intervention will be conducted. Intensity: The subject’s heart rate should be controlled between 55% and 65% HR_max_. If it exceeds the controlled heart rate, the subject’s exercise intensity should be appropriately reduced.	Do not engage in any other forms of exercise except for maintaining past lifestyle habits.	60	3	48
Cheng, 2022 [10]	RCT	Exp—N: 24 Con—N: 25 Exp—Age: 50.2 ± 3.1 Con—Age: 50.1 ± 2.9	Tai Chi Quan	Perimenopause (45–55 years), defined by menopausal symptoms (e.g., hot flashes, sweating, and irritability) and a Kupperman index < 30. Participants were further described as postmenopausal, premenopausal, or having menopausal disorder, but no operational definitions or formal reproductive staging criteria were provided.	A 24-style simplified form of TC was selected for the TC group. The TC exercises have a fixed voice prompt, including 24 movements (∼5 min is required to complete the exercise), and each movement completion time is fixed, for which the exercise rate is fixed.	Only the Tai Chi and control arms were included in the present review; the brisk-walking arm was excluded because it did not meet the predefined mind–body exercise criterion.	60	3	48
Xiao, 2015 [40]	RCT	Exp—N: 20 Con—N: 20 Age: 55.5	Tai Chi Rouli ball	Perimenopause (mean age 55.5 years); participants were explicitly described as perimenopausal by the original study, but no operational criteria for menopausal staging were provided.	The Tai Chi group practiced Tai Chi Rouli ball for 6 months.	The control group received no intervention.	90	3	26
Xin, 2024 [42]	RCT	Exp—N: 27 Con—N: 25 Exp—Age: 51.15 ± 3.32 Con—Age: 50.93 ± 3.66	Tai Chi Rouli ball	Perimenopause (eligibility age 45–55 years), defined by irregular menstrual cycles without having reached full postmenopause, together with estradiol levels > 30 pg/mL and FSH levels < 40 IU/L. No formal reproductive staging system was reported.	Participants in the Tai Chi Rouli Ball group engaged in the exercise regimen three times a week for a period of six months. Intensity: moderate (was quantitatively assessed using the Borg Rating of Perceived Exertion scale).	The control group kept weekly self-reported physical activity diaries, which were used to monitor any major changes in their usual routines that might influence the study outcomes.	60	3	26
Liu, 2024 [39]	RCT	Exp—N: 15 Con—N: 15 Exp—Age: 53.33 ± 2.36 Con—Age: 53.87 ± 1.31	Hatha yoga	Perimenopause (45–55 years), defined primarily by 0–12 months of natural amenorrhea, with menopause-related symptoms also considered. The study provided conceptual criteria for early and late perimenopause but did not formally assign participants to specific reproductive stages or use endocrine staging criteria.	The yoga exercise program is divided into three parts: preparation, basic exercises, and conclusion. The exercise intensity is measured by the average heart rate of the participants. The preparation part lasts for 5 to 8 min, with a moderate exercise intensity. The basic exercises part lasts for 40 to 50 min, with a higher exercise intensity. The conclusion part lasts for 5 to 8 min, with a lower exercise intensity.	The control group only participated in general physical exercise activities and daily activities according to the state of daily life, and the exercise time and frequency were the same as those of the experimental group.	60	3	12
Gu, 2016 [44]	RCT	Exp—N: 39 Con—N: 39 Exp—Age: 50.84 ± 3.09 Con—Age: 49.74 ± 3.32	Wu Xing Jian Gu Cao	Perimenopause (45–55 years), staged according to the STRAW system: early menopausal transition was defined by ≥2 changes in menstrual cycle length of >7 days within the previous 10 cycles; late menopausal transition by amenorrhea for ≥60 days or more than two menstrual cycles, with possible vasomotor symptoms; and early postmenopause by <1 year since the final menstrual period.	Wu Xing Jian Gu Cao exercise: It is stipulated that the exercise guidance of five element bone exercises should be carried out twice a week, and there should be one self-exercise in the rest of the time, each time for 30–45 min, to ensure that the exercise can be completely practiced twice. Degree of exercise intervention: the amount of exercise is mainly based on the subjects’ adaptability (refer to the appropriate exercise heart rate = 170− age/min)	The implementation methods of health education prescription include: issuing Disease Knowledge Manual; two centralized lectures per month (physicians and senior nurses will give lectures on the popularization of disease knowledge and guide the subjects’ correct healthy lifestyle through PPT presentation and group discussion)	37.5	3	26

Note: N, sample size; Fre, training frequency (sessions/week); Wk, training intervention weeks; Dur, single exercise duration; RCT, randomized controlled trial; CT, non-randomized controlled trials; NR, not reported; HR, heart rate; Exp, experimental; Con, control.
