# Supplementary material for: Effects of Mind–Body Exercise on Bone Health in Perimenopausal Women: A Systematic Review and Three-Level Meta-Analysis
Source: Life (Basel). 2026 Aug 23;16(9):1389. doi: 10.3390/life16091389 (PMC13608597; doi:10.3390/life16091389)
Supplement: Supplementary file 1 [file life-16-01389-s001.zip › life-4447661-supplementary.pdf]

Supplementary Materials Table S1. Prisma Checklist 2020

Section and Topic	Item #	Checklist item	Location where item is reported
**TITLE**			
Title	1	Identify the report as a systematic review.	1
**ABSTRACT**			
Abstract	2	See the PRISMA 2020 for Abstracts checklist.	1
**INTRODUCTION**			
Rationale	3	Describe the rationale for the review in the context of existing knowledge.	2
Objectives	4	Provide an explicit statement of the objective(s) or question(s) the review addresses.	3
**METHODS**			
Eligibility criteria	5	Specify the inclusion and exclusion criteria for the review and how studies were grouped for the syntheses.	3-4
Information sources	6	Specify all databases, registers, websites, organisations, reference lists and other sources searched or consulted to identify studies. Specify the date when each source was last searched or consulted.	3;20-21
Search strategy	7	Present the full search strategies for all databases, registers and websites, including any filters and limits used.	20-21
Selection process	8	Specify the methods used to decide whether a study met the inclusion criteria of the review, including how many reviewers screened each record and each report retrieved, whether they worked independently, and if applicable, details of automation tools used in the process.	3
Data collection process	9	Specify the methods used to collect data from reports, including how many reviewers collected data from each report, whether they worked independently, any processes for obtaining or confirming data from study investigators, and if applicable, details of automation tools used in the process.	4
Data items	10a	List and define all outcomes for which data were sought. Specify whether all results that were compatible with each outcome domain in each study were sought (e.g. for all measures, time points, analyses), and if not, the methods used to decide which results to collect.	3-4
	10b	List and define all other variables for which data were sought (e.g. participant and intervention characteristics, funding sources). Describe any assumptions made about any missing or unclear information.	4-5
Study risk of bias assessment	11	Specify the methods used to assess risk of bias in the included studies, including details of the tool(s) used, how many reviewers assessed each study and whether they worked independently, and if applicable, details of automation tools used in the process.	5
Effect measures	12	Specify for each outcome the effect measure(s) (e.g. risk ratio, mean difference) used in the synthesis or presentation of results.	6
Synthesis methods	13a	Describe the processes used to decide which studies were eligible for each synthesis (e.g. tabulating the study intervention characteristics and comparing against the planned groups for each synthesis (item #5)).	3-5
	13b	Describe any methods required to prepare the data for presentation or synthesis, such as handling of missing summary statistics, or data conversions.	4-5
	13c	Describe any methods used to tabulate or visually display results of individual studies and syntheses.	6, 8-13, 23-31
	13d	Describe any methods used to synthesize results and provide a rationale for the choice(s). If meta-analysis was performed, describe the model(s), method(s) to identify the presence and extent of statistical heterogeneity, and software package(s) used.	5-6
	13e	Describe any methods used to explore possible causes of heterogeneity among study results (e.g. subgroup analysis, meta-regression).	6
	13f	Describe any sensitivity analyses conducted to assess robustness of the synthesized results.	6, 12-13

Section and Topic	Item #	Checklist item	Location where item is reported
Reporting bias assessment	14	Describe any methods used to assess risk of bias due to missing results in a synthesis (arising from reporting biases).	6, 23-25
Certainty assessment	15	Describe any methods used to assess certainty (or confidence) in the body of evidence for an outcome.	6
**RESULTS**			
Study selection	16a	Describe the results of the search and selection process, from the number of records identified in the search to the number of studies included in the review, ideally using a flow diagram.	6, 7
	16b	Cite studies that might appear to meet the inclusion criteria, but which were excluded, and explain why they were excluded.	7
Study characteristics	17	Cite each included study and present its characteristics.	7
Risk of bias in studies	18	Present assessments of risk of bias for each included study.	7-8
Results of individual studies	19	For all outcomes, present, for each study: (a) summary statistics for each group (where appropriate) and (b) an effect estimate and its precision (e.g. confidence/credible interval), ideally using structured tables or plots.	8-12, 26-31
Results of syntheses	20a	For each synthesis, briefly summarise the characteristics and risk of bias among contributing studies.	7-12
	20b	Present results of all statistical syntheses conducted. If meta-analysis was done, present for each the summary estimate and its precision (e.g. confidence/credible interval) and measures of statistical heterogeneity. If comparing groups, describe the direction of the effect.	8-12; 26-31
	20c	Present results of all investigations of possible causes of heterogeneity among study results.	8-12
	20d	Present results of all sensitivity analyses conducted to assess the robustness of the synthesized results.	12-13, 31
Reporting biases	21	Present assessments of risk of bias due to missing results (arising from reporting biases) for each synthesis assessed.	7, 23-25
Certainty of evidence	22	Present assessments of certainty (or confidence) in the body of evidence for each outcome assessed.	13-14
**DISCUSSION**			
Discussion	23a	Provide a general interpretation of the results in the context of other evidence.	14-15
	23b	Discuss any limitations of the evidence included in the review.	16
	23c	Discuss any limitations of the review processes used.	16
	23d	Discuss implications of the results for practice, policy, and future research.	15-17
**OTHER INFORMATION**			
Registration and protocol	24a	Provide registration information for the review, including register name and registration number, or state that the review was not registered.	3
	24b	Indicate where the review protocol can be accessed, or state that a protocol was not prepared.	3
	24c	Describe and explain any amendments to information provided at registration or in the protocol.	N/a
Support	25	Describe sources of financial or non-financial support for the review, and the role of the funders or sponsors in the review.	17
Competing interests	26	Declare any competing interests of review authors.	17

Section and Topic	Item #	Checklist item	Location where item is reported
Availability of data, code and other materials	27	Report which of the following are publicly available and where they can be found: template data collection forms; data extracted from included studies; data used for all analyses; analytic code; any other materials used in the review.	n/a

From: Page MJ, McKenzie JE, Bossuyt PM, Boutron I, Hoffmann TC, Mulrow CD, et al. The PRISMA 2020 statement: an updated guideline for reporting systematic reviews. BMJ 2021;372:n71. doi: 10.1136/bmj.n71

For more information, visit: http://www.prisma-statement.org/

Supplementary Materials Table S2. Rob2 judgement reason

Study	Domain	Judgement	Reason
Du, 2014	D1	Some concerns	The study reported random allocation, but the method of sequence generation and allocation concealment was not described. No important baseline imbalance suggesting failure of randomization was identified.
	D2	Low risk	Participants and intervention personnel were aware of intervention assignment because blinding was not feasible for the exercise intervention. Although no specific deviations from the intended interventions were reported, information on intervention adherence, contamination in the control group, and other co-interventions during the 24-week intervention was insufficient to rule out deviations arising from the trial context.
	D3	Low risk	Outcome data were available for all 30 participants, with 15 participants in each group at baseline and the same sample sizes reported for post-intervention BMD and BMC analyses.
	D4	Low risk	BMD and BMC were objectively measured using the same DXA equipment and assessment schedule in both groups. Although assessor blinding was not reported, knowledge of intervention assignment was unlikely to materially influence the objectively measured outcomes.
	D5	Some concerns	No prospective protocol or prespecified statistical analysis plan was available, and the study did not identify a primary BMD/BMC outcome or anatomical site. Multiple eligible outcome measurements across different skeletal sites and different statistical comparisons were reported. Therefore, although results for several nonsignificant outcomes were also presented, selection of the reported result based on the observed findings could not be fully ruled out.
Zhao, 2020	D1	Some concerns	The study reported numerical random allocation, but details of sequence generation and allocation concealment were not provided. No important baseline imbalance suggesting a failure of randomization was observed.
	D2	Some concerns	Participants and intervention personnel were aware of group assignment because blinding was not feasible. Although both groups were regularly followed to monitor additional exercise, diet, and calcium/vitamin D intake, quantitative information on adherence to the prescribed 48-week Tai Chi intervention and actual deviations from the assigned interventions was not reported. Therefore, deviations arising from the trial context could not be fully ruled out.
	D3	Low risk	All 74 randomized participants completed the study and were included in the outcome analyses. The 12 withdrawals occurred before random allocation and therefore did not result in missing outcome data among randomized participants.
	D4	Low risk	BMD was objectively assessed using the same dual-energy X-ray absorptiometry procedure in both groups, and the study explicitly reported blinded outcome assessment. Knowledge of intervention assignment was therefore unlikely to influence BMD measurement.

	D5	Some concerns	Although the prespecified outcome section identified lumbar spine and proximal femur BMD and all measured skeletal sites were reported, no prospective trial registration or prespecified statistical analysis plan was available for verification. Given multiple eligible BMD measurements and statistical comparisons, selection of the reported result based on the observed findings could not be fully ruled out.
Cheng, 2022	D1	Some concerns	The study reported random allocation to the Tai Chi, brisk walking, and control groups, but the method used to generate the allocation sequence and allocation concealment procedures were not described. No important baseline imbalance was observed.
	D2	Low risk	Although participants and intervention personnel were aware of group assignment, the intervention was standardized and researchers conducted weekly follow-up of all groups to monitor additional exercise, dietary habits, sunlight exposure, and medications affecting BMD. No important deviations from the intended interventions attributable to the trial context were reported.
	D3	Some concerns	The participant flow diagram indicated that 91 participants were randomized, but only 75 were included in the final analysis, corresponding to approximately 17.6% post-randomization attrition. Although attrition was relatively balanced across groups, the reasons for some losses, including voluntary withdrawal and inability to contact participants, were insufficient to exclude a relationship between missingness and the true outcome.
	D4	Low risk	BMD was objectively measured using the same dual-energy X-ray absorptiometry system, anatomical sites, and assessment schedule across all groups. The objective nature of BMD measurement makes substantial influence of knowledge of intervention assignment unlikely.
	D5	Some concerns	The study included multiple eligible BMD outcomes across four skeletal sites, four assessment timepoints, and several within- and between-group statistical comparisons. Although both significant and nonsignificant results were reported, no independently verifiable prespecified analysis plan identifying the primary BMD result was available, so selection from multiple eligible results could not be fully ruled out.
Xiao, 2015	D1	Some concerns	The study stated that participants were randomized into two groups, but the method of random sequence generation and allocation concealment was not described. No clear evidence of substantial baseline imbalance suggesting failure of randomization was identified.
	D2	Some concerns	Participants and intervention personnel were necessarily aware of group assignment. The study provided little information on adherence to the 6-month Tai Chi Ball intervention, contamination in the control group, or other co-interventions that could affect bone outcomes. Therefore, deviations from the intended interventions arising from the trial context could not be fully ruled out.
	D3	Low risk	Forty participants were randomized, with 20 participants assigned to each group, and baseline and post-intervention outcome data were reported for both groups. No participant attrition or missing outcome data were reported.
	D4	Low risk	BMD and BMC were objective bone outcomes measured at predefined anatomical sites at baseline and 6

			months in both groups. Although details of assessor blinding and the measurement device were not reported, there was no evidence that outcome measurement differed between groups or that knowledge of intervention assignment was likely to materially influence the measurements.
	D5	Some concerns	No prospective trial registration or prespecified statistical analysis plan was available, and no primary BMD/BMC outcome or anatomical site was clearly identified. Multiple eligible bone outcome measurements and statistical comparisons were available. Although both significant and nonsignificant results were presented, selection of the reported result based on the observed findings could not be fully ruled out.
Xin, 2024	D1	Low risk	Participants were randomized using computer-generated random numbers, and no important baseline imbalances suggesting a problem with the randomization process were observed.
	D2	Low risk	The intervention was standardized and adherence was actively monitored using a digital platform, with an average attendance rate of 87%. Physical activity in the control group was also monitored, and no important deviations from the intended interventions attributable to the trial context were reported.
	D3	Some concerns	Although 52 participants were reported in the main study results and the final group sizes accounted for all 52 participants, the limitation section inconsistently referred to a sample size of 60 participants. Because participant flow and any potential post-randomization exclusions were not clearly documented, the completeness of outcome data could not be fully verified.
	D4	Low risk	BMD and BMC were objectively measured using the same Hologic Discovery A DXA system and standardized procedures before and after the intervention, making differential outcome measurement unlikely.
	D5	Low risk	BMD and BMC were explicitly identified as primary outcomes, and results across all measured anatomical sites were reported, including both statistically significant and nonsignificant findings. There was no clear evidence that the reported BMD/BMC results were selected on the basis of the observed findings.
Liu, 2024	D1	Some concerns	Participants were reported to be randomly assigned to the intervention and control groups, but the method of random sequence generation and allocation concealment was not described. No important baseline imbalance suggesting failure of randomization was identified.
	D2	Some concerns	Participants and intervention personnel were aware of group assignment. Although the Hatha yoga intervention was supervised and participants were instructed to avoid additional exercise and maintain their usual diet, quantitative information on attendance, adherence, and potential co-interventions during the 12-week intervention was not reported. Therefore, deviations from the intended interventions could not be fully ruled out.
	D3	Low risk	All 30 participants, including 15 in each group, completed both pre- and post-intervention assessments, and the study explicitly reported that the sample size remained unchanged throughout the experiment.
	D4	Some	Bone outcomes were assessed using DXA; however, the reported “BMD” values were negative and were

		concerns	labelled as g/cm², while the methods described T-score diagnostic thresholds. This creates uncertainty as to whether the reported outcome represents absolute BMD or T-scores, although the same measurement approach appears to have been applied to both groups.
	D5	Some concerns	The study assessed multiple bone density outcomes (hip, ankle, and whole-body measurements) and used both within-group and between-group statistical comparisons. No prospective trial registration or independently verifiable prespecified statistical analysis plan was reported, and a primary bone density outcome or primary analysis was not clearly identified. Therefore, selection of the reported result from multiple eligible measurements or analyses could not be fully ruled out.
Gu, 2016	D1	Some concerns	A random number table was used to generate the allocation sequence, but allocation concealment and the implementation of random assignment were not described. No important baseline imbalance suggesting failure of randomization was identified.
	D2	Some concerns	Participants and intervention personnel were aware of group assignment. Although the intervention was standardized and exercise logs were required under family supervision, quantitative information on actual adherence, completion of self-directed exercise sessions, and potential contamination or other co-interventions during the 6-month intervention was not reported. Therefore, deviations from the intended interventions could not be fully ruled out.
	D3	Low risk	Outcome data were available for 78 of 80 randomized participants (97.5%). Only two participants were lost, with one loss in each group, resulting in minimal and balanced missing outcome data.
	D4	Low risk	Bone metabolism outcomes were objectively assessed using standardized biochemical and chemiluminescence assays at baseline and 6 months, with the same measurement procedures applied to both groups. Knowledge of intervention assignment was unlikely to materially influence these laboratory-based outcomes.
	D5	Low risk	The bone metabolism outcomes assessed in the study were specified in the Methods, and the corresponding results were reported comprehensively, including both statistically significant and nonsignificant findings. For each specific bone metabolism outcome, there was no clear evidence of selection from multiple eligible measurements or analyses based on the observed results.

Note: D1, Bias arising from the randomization process; D2, Bias due to deviations from intended intervention; D3, Bias due to missing outcome data; D4, Bias in measurement of the outcome; D5, Bias in selection of the reported result.
